# Adverse childhood experiences, adult adiposity, and risk of young-onset breast cancer subtypes in a population-based case-control study

**DOI:** 10.1007/s10552-026-02171-y

**Published:** 2026-05-26

**Authors:** Lydia Marcus Post, Young Ik Cho, James Topitzes, DPhuong Do, Dorothy R. Pathak, Ann S. Hamilton, Amani M. Nuru-Jeter, Kelly A. Hirko, Jamila L. Kwarteng, Richard Houang, Ann G. Schwartz, Ellen M. Velie

**Affiliations:** 1 Epidemiology Program, Joseph J. Zilber College of Public Health, University of Wisconsin–Milwaukee, Milwaukee, WI 53205 USA; 2 Community & Behavioral Health Promotion Program. Joseph J. Zilber College of Public Health, University of Wisconsin–Milwaukee, Milwaukee, WI USA; 3 Social Work Department, Helen Bader School of Social Welfare, University of Wisconsin–Milwaukee, Milwaukee, WI USA; 4 Public Health Policy Program, Joseph J. Zilber College of Public Health, University of Wisconsin–Milwaukee, Milwaukee, WI USA; 5 Department of Epidemiology and Biostatistics, College of Human Medicine, Michigan State University, East Lansing, MI USA; 6 Department of Population and Public Health Sciences, Keck School of Medicine, University of Southern California, Los Angeles, CA USA; 7 Divisions of Community Health Sciences & Epidemiology, School of Public Health, University of Californiaa–Berkley, Berkeley, CA USA; 8 Division of Community Health, Institute for Health and Humanity, Medical College of Wisconsin, Milwaukee, WI USA; 9 Center for the Study of Curriculum, College of Education, Michigan State University, East Lansing, MI USA; 10 Department of Oncology, School of Medicine, Wayne State University and the Karmanos Cancer Institute, Detroit, MI USA; 11 Departments of Medicine and Pathology, Medical College of Wisconsin, Milwaukee, WI USA

**Keywords:** Young onset breast cancer, Tumor subtypes, Adverse childhood experiences, Adult adiposity

## Abstract

**Purpose:**

Adverse childhood experiences (ACEs) are hypothesized to increase breast cancer risk via social and physiological pathways, yet associations between ACEs and young-onset breast cancer (BC) subtypes–and potential mediators (e.g., adiposity) of these associations–are largely unstudied. We sought to evaluate associations between ACEs and young-onset BC molecular subtypes, and the potential mediating effects of adiposity, in a population-based case–control study of invasive young-onset BC.

**Methods:**

Cases (n = 1,754) were identified from the Los Angeles County and Metropolitan Detroit SEER registries, 2010–2015, and area-based, frequency-matched controls (n = 1,350) sampled from the 2010 Census. Associations between ACEs aged < 18 years and young-onset BC (diagnosed ages 20 to < 50 years), overall and by subtype (luminal A, luminal B, HER2 + , triple negative [TN]), were examined using weighted logistic regression. Adult adiposity (i.e., body mass index 12 months before diagnosis, waist circumference at interview) was evaluated as a mediator of these associations using weighted path analyses.

**Results:**

ACEs (≥ 1 vs 0) were not significantly associated with young-onset BC (all p_trend_ > 0.10); however, reporting one ACE (vs 0) was associated with TN (odds ratio [OR] = 1.85 [95% confidence interval [95%CI] = 1.08–3.16]). For individual ACEs, *death of a loved one* (vs 0 ACEs) was associated with HER2 + (OR = 2.50 [95%CI = 1.06–5.88]) and potentially TN (OR = 1.72 [95%CI = 0.96–3.10]) young-onset BC; other associations were non-significant. In path analyses, ACEs had a significant total effect on luminal A (β = 0.08, p = 0.03) and TN (β = 0.13, p < 0.01) tumors, with adiposity mitigating this association for luminal A tumors (β = -0.01, p = 0.04; TN β = 0.00, p > 0.05).

**Conclusions:**

Bereavement during childhood was associated with higher odds of HER2 + young-onset BC, and there was some evidence of an association between ACEs and other young-onset BC subtypes, particularly TN.

**Supplementary Information:**

The online version contains supplementary material available at 10.1007/s10552-026-02171-y.

## Introduction

Breast cancer is the most commonly diagnosed cancer, besides nonmelanoma skin cancers, and is the leading cause of cancer death among young women in the United States [1, 2]. The incidence of young-onset breast cancers (BCs; i.e., diagnosed < 50 years of age) is increasing [3]; however, the etiology of young-onset BC, particularly by tumor subtypes, is poorly understood [4–6]. Additionally, young non-Hispanic Black compared to White women face a significantly higher risk of developing aggressive hormone receptor (HR; i.e., estrogen receptor [ER], progesterone receptor [PR])-negative tumors and are nearly twice as likely to die from young-onset BC [2]. The limited existing evidence also suggests that poorer women face a similarly elevated risk of HR-negative BC diagnosed at any age [7, 8]. Adverse life experiences, which are more common among non-Hispanic Black populations and those experiencing poverty [9, 10], may increase BC risk [7, 11–13] and contribute to young-onset BC disparities. Furthermore, adversities experienced in childhood (i.e., adverse childhood experiences, ACEs)—a sensitive period for breast development [14, 15]—may have particular relevance for subsequent BC risk [16–18].

Previous work evaluating the mechanisms hypothesized to underly associations between adversity and breast cancer suggests that experience of adversities causes dysregulation of cortisol secretion via psychosocial stress pathways [13, 19–22]. This indirectly increases estrogen production, alters breast tissue, and downregulates the expression of tumor suppressor genes [13, 19–21]. Through similar stress pathways, ACEs are also associated with increased risk of outcomes like general and central adiposity [21, 23, 24]. Central adiposity, in turn, is associated with elevated risk for aggressive young-onset BC tumors (i.e., triple negative [TN; HR-negative, HER2-negative] [25–29], and potentially the HR-positive luminal B subtype [29]). General adiposity, as measured by body mass index (BMI), however, is consistently associated with reduced risk of young-onset BC overall, and the less aggressive HR-positive luminal A molecular subtype in particular [30, 31].

To our knowledge, no previous studies have evaluated associations between early life adversities and young-onset BC molecular subtypes. Eight previous studies examined associations between childhood adversity and BC risk [7, 11, 12, 32–36]. Of these, only three considered risk by tumor types (i.e., ER status [7, 11, 36]; PR status [36]; HER2 status [36]), which evidence suggests have distinct etiologies [37–40]. BC etiology is also thought to vary by age at diagnosis, yet only one study evaluated associations between ACEs and young-onset BC overall (not by subtype) [11] and one evaluated associations with a single childhood adversity (parental death) and young-onset BC tumor type (diagnosed by age 52 years)[36]. Findings from these studies suggest a positive association between some childhood adversities (e.g., *sexual abuse*, *household substance abuse*, *parental death*) and both ER-negative and ER-positive BC [7, 11, 36], as well as young-onset BC overall [11], though few associations reached statistical significance, potentially due to limited sample size for rare BC outcomes.

Thus, we sought to contribute to the current understanding of ACEs (both individually and in total) and risk of young-onset BC overall and by tumor subtype in a socioeconomically diverse, population-based sample of non-Hispanic Black and non-Hispanic White young women. We hypothesized that ACEs would be associated with increased young-onset BC risk, both because of the physiological changes induced by ACEs during an early stage of breast development and because evidence suggests that childhood adversity shapes future exposure to BC risk factors, such as adult adiposity [18, 22, 29]. Because ACEs have been associated with increased waist circumference and we have previously observed a strong positive association between waist circumference and TN young-onset BC [29], and a weaker positive association with luminal B young-onset BC [29], we additionally explored whether associations between ACEs and certain young-onset BC subtypes were partially mediated by adult adiposity. To our knowledge this is the first analysis to evaluate associations between ACEs and young-onset BC by tumor molecular subtype in a population-based sample.

## Methods

### Study population

Data are from the Young Women’s Health History Study (YWHHS), which is described elsewhere in detail [41]. Briefly, YWHHS is a population-based case–control study conducted among US-born non-Hispanic Black and White women aged 20–49 years residing in los angeles (LA) County and Metropolitan Detroit (Oakland, Wayne, and Macomb Counties). Additional eligibility criteria included that participants were able to complete the in-person interview in English, were not institutionalized at their reference date (i.e., date of diagnosis for cases; four months prior to screening for inclusion in YWHHS for controls), had no previous cancer diagnosis (excepting cervical in situ or common skin cancer), and were well enough to complete the interview [41]. Cases were diagnosed with histologically confirmed invasive BC in LA County and Metropolitan Detroit Surveillance, Epidemiology, and End Results (SEER) registries 2010–2015 (N = 1812; n = 682 non-Hispanic Black, n = 1,130 NHW non-Hispanic White. Controls were identified from over 24,000 potentially eligible households via three-stage area-based probability sampling from the 2010 Census and frequency-matched to cases on site (LA/Detroit), five-year age intervals, and race (non-Hispanic Black/non-Hispanic White) (N = 1,381; n = 665 non-Hispanic Black, n = 716 non-Hispanic White). Response rates were 60% for cases and 53% for controls [42]; non-response weights incorporate information from demographic data available from 86% of sampled controls and 100% of sampled cases [41]. YWHHS sample characteristics have been described previously; distribution of the characteristics used for frequency matching (i.e., study site, age, and race) are similar by case/control status [41].

The YWHHS protocol was approved by the Institutional Review Boards (IRBs) at University of Wisconsin–Milwaukee (UWM), Milwaukee, WI (Medical College of Wisconsin deferred to UWM); Michigan State University, East Lansing, MI; Wayne State University, Detroit, MI; Michigan Department of Community Health, MI; University of Southern California, LA, CA; California Committee for the Protection of Human Subjects, CA; and California Cancer Registry. All study participants provided written informed consent.

### Outcome assessment

Histopathologic diagnosis of invasive breast tumors, tumor grade, and expression of ER, PR, and HER2 were collected by the LA County Cancer Surveillance Program and Metropolitan Detroit Cancer Surveillance System SEER registries and provided to YWHHS [43].

*Tumor molecular subtypes.* Tumors were classified as luminal A (ER-positive and/or PR-positive and HER2-negative and grade 1/2); luminal B (either ER-positive and/or PR-positive and HER2-positive or ER-positive and/or PR-positive and HER2-negative with grade 3); HER2 + (ER-negative, PR-negative, and HER2-positive); or TN (ER-negative, PR-negative, and HER2-negative).

### Exposure and covariate assessment

Self-reported ACEs, adiposity, and covariates were ascertained via an in-person computer assisted interview with life history calendars to facilitate recall [44]. Current height, weight, and waist circumference were measured during the interview by trained interviewers [29].

#### Adverse childhood experiences

Participants were asked about possible exposure before age 13 years to the following 12 ACEs: if their parent or primary childhood caregiver experienced a serious illness, substance abuse, imprisonment, or separation/divorce; whether they themselves experienced the death of a loved one, negative interactions with the police, domestic violence in their home, discrimination (from any source), vicarious discrimination (i.e., witnessing a loved one experience discrimination), physical abuse, verbal abuse, or sexual abuse[45]. Participants were additionally asked about exposure to sexual abuse before age 18 years (Table S1). Most ACE questions had binary responses (ever/never); the questions regarding *domestic violence*, *physical abuse*, and *verbal abuse* had multilevel responses (i.e., never, 1, 2–5, 6–10, 11–20, 21–50, or ≥ 50 times) and were collapsed into a binary response (ever/never) for analysis, to facilitate creation of an ACE index and improve comparability with prior studies of ACEs (Table S1). For the ACEs personal experiences of discrimination (hereafter “*personal discrimination”*) and *vicarious discrimination*, participants reported the frequency of their experiences in multiple settings (e.g., in public, at school, in situations with police or legal authorities). We previously used weighted latent class analysis to identify two levels of discrimination exposure, “higher” and “lower,” where participants with “lower” exposure were unlikely to report discrimination in any setting[45]. Most ACE questions were adapted from the Behavioral Risk Factor Surveillance System ACE questionnaire [46] and assessment of childhood discrimination experiences was adapted from a measure previously developed by collaborator TPD [47]. ACEs were evaluated individually and as an index (i.e., number of ACEs experienced, range 0–12), categorized as 0/≥ 1 and 0/1/2/≥ 3, to represent levels of ACE exposure (i.e., none/below median/median/above median). The ACEs *caregiver incarceration* and *negative police interactions* were combined as *police contact* for analyses of individual ACEs due to small sample size.

#### Adult adiposity

Waist circumference was measured at interview and BMI 12 months before reference date (hereafter “adult BMI”) was calculated from “corrected” recalled weight and measured height. Recalled weight 12 months before reference date was corrected by applying the ratio of self-reported to measured weight at interview (correlation r = 0.98 for cases and r = 0.96 for controls, respectively) [29]. If measured or recalled weight at interview was unavailable (n = 105), uncorrected recalled weight was used to calculate adult BMI, and if measured height was unavailable (n = 157), recalled adult height was used (r = 0.95 and r = 0.94, respectively) [29].

#### Childhood household socioeconomic position

A childhood household socioeconomic position (SEP) index was previously created [45] using weighted polychoric principal component analysis with varimax rotation [48, 49]. Childhood household SEP indicators included how often before age 13 years the participant’s household received government assistance, lacked money for essentials (e.g., rent or mortgage, electricity), lacked a reliable car, or experienced food insecurity. One identified principal component had an eigenvalue of ≥ 1.0, and this component was used as the childhood household SEP index; factors loaded similarly on this component [45].

#### Race

Participants who selected either “Black/African American” or “White” as the race they most identified with and who did not identify as Hispanic/Latina were eligible for inclusion in YWHHS and were categorized as non-Hispanic Black or non-Hispanic White [41].

Additional covariates included age at reference date, study site, and first-degree family history of BC. Other established BC risk factors that are associated with ACEs were not included as covariates [50–54], as they could potentially mediate associations between ACEs and young-onset BC.

### Missingness

Cases who were missing tumor pathology data on receptor status and/or tumor grade were omitted from young-onset BC subtype analyses (n = 126 total, 7%) but included in analyses of young-onset BC overall. Participants missing data on at least one ACE (n = 58 cases; n = 31 controls) were excluded from all analyses. These participants tended to have lower childhood household SEP but were otherwise similar in sociodemographic factors and adiposity to participants with complete ACE data (Table S2). No participants lacked data on age, study site, or race. Participants who did not know or were missing information on first-degree family history of BC (n = 135) were categorized as “don’t know” and retained in analyses. Participants missing data on at least one childhood household SEP indicator (n = 42 cases; n = 39 controls) were excluded from adjusted logistic regression models and path analysis equations including childhood household SEP. Non-Hispanic Black women were more likely than non-Hispanic White women to lack childhood household SEP data (4.4 vs 1.1% missing among cases and 3.9% vs 1.4% missing among controls, respectively; p ≤ 0.01). Participants missing data on adult BMI (n = 34 cases; n = 38 controls) or waist circumference (n = 87 cases; n = 56 controls) were excluded from path analysis equations including BMI or waist circumference, respectively.

### Statistical analyses

We used weighted logistic regression to calculate the odds ratios and 95% confidence intervals (OR [95%CIs]) of young-onset BC, overall and by subtype, associated with reporting at least one ACE (≥ 1 vs 0), different levels of ACE exposure (1, 2, ≥ 3 vs 0), and for individual adversities (vs 0). We also tested for linear trend considering ACE index as a continuous variable. The minimally adjusted model included age (continuous), study site (Detroit/LA), and first-degree family history of BC (yes/no/don’t know). The fully adjusted model additionally included childhood household SEP index (continuous) and race (non-Hispanic Black/non-Hispanic White), two factors that shape risk of ACEs [45, 55, 56] and potentially also young-onset BC [2, 7, 57, 58]. Besides the three characteristics used for frequency matching (i.e., study site, age, and race), distributions of these other covariates varied by case/control status. We assessed the heterogeneity of associations between ACEs and young-onset BC subtypes using multinomial logistic regression and Wald tests of significance. To facilitate comparison with other studies of ACEs, which typically omit experiences of discrimination, we also conducted sensitivity analyses omitting the two discrimination ACEs from the ACE index (range then 0–10). As a supplemental analysis, we evaluated effect modification by first degree family history of breast cancer (yes/no) using likelihood ratio tests of cross-product terms. Additionally, as a sensitivity analysis, we evaluated associations with luminal-type young-onset BC defined according to hormone receptor status alone (i.e., HR +/HER2− and HR +/HER2 +; all luminal A tumors and most luminal B tumors are HR +/HER2−).

**Fig. 1:**
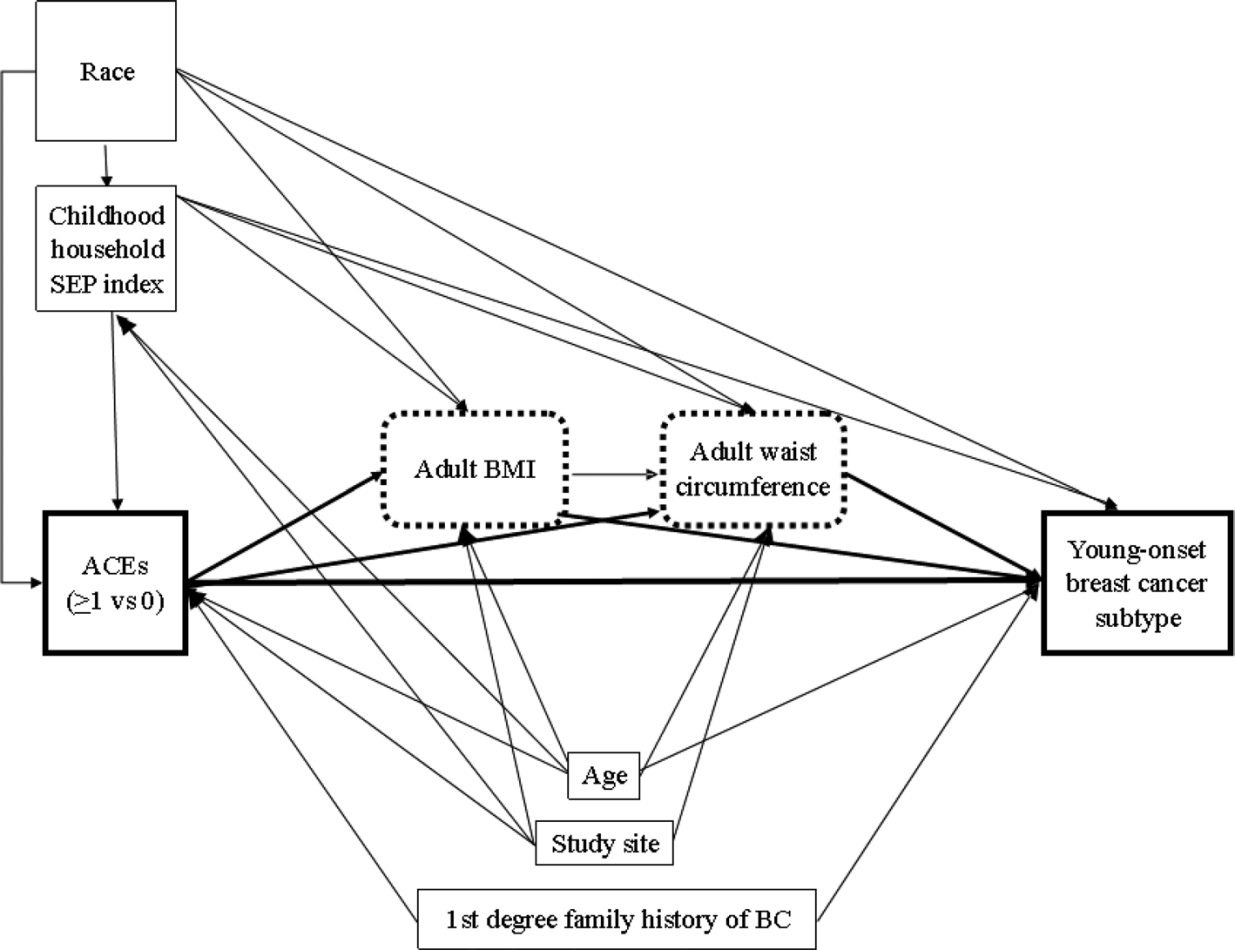
Path model evaluating the association between Adverse Childhood Experiences (ACEs) and young onset breast cancer (BC) subtypes and potential mediation by body size *Note*: Path analysis modeling the association of ACEs (0/≥ 1) with young-onset BC subtype risk (luminal A, luminal B, and TN each vs controls). Covariates included study site (Detroit/LA), age (years), first degree family history of breast cancer (yes/no/don’t know), race (non-Hispanic Black/non-Hispanic White), childhood household SEP index (continuous index created using polychoric principal component analysis from SEP indicators aged < 13 years), recent adult body mass index (BMI; kg/m^2^, continuous), and measured adult waist circumference (cm, continuous)

We also conducted exploratory path analyses to evaluate whether adult adiposity (waist circumference, in cm, and BMI, in kg/m^2^; both continuous) mediated associations between ACEs and select young-onset BC subtypes (luminal A, luminal B, and TN; Fig. 1). Models were not conducted for HER2 + tumors due to limited sample size. We parameterized the ACEs index as ≥ 1 versus 0. We included childhood household SEP and race as factors that potentially affect risk of ACEs, adult adiposity, and young-onset BC; we included study site, age, and first-degree family history of BC as potential confounders (Fig. 1). We used the weighted least square mean and variance adjusted (WLSMV) estimator, which does not assume normally distributed variables [59]. Model selection was based on the standardized root mean square residual (SRMR), with values < 0.08 indicating acceptable model fit.[59] The final models for the three subtypes had identical specifications and demonstrated excellent fit (SRMR ≤ 0.02).

All analyses incorporate sample weights to reflect probability of selection, ensure representativeness of participants to the Metropolitan Detroit and LA County study populations and address non-response [41]. Path analyses were performed in Mplus Version 8 (Muthen & Muthen, LA, CA). All other analyses were performed in Stata version 16.1 (StataCorp LLC, College Station, TX).

## Results

In our population-based sample of young non-Hispanic Black and non-Hispanic White women, the distribution of ACE index values were similar between cases and controls, though higher ACE index was somewhat more common among controls than cases (i.e., ≥ 3 ACEs 48.1 vs 46.3%, respectively) and highest among HER2 + cases (54.7%) (Table 1). There was, however, some variation in individual ACEs by case/control status. Among controls, *verbal abuse* was the most reported ACE, whereas, among cases, *personal discrimination* was the most reported. By cancer subtype, *caregiver substance abuse* and *verbal abuse* were most common for women with luminal A (25.9 and 36.2%, respectively) and HER2 + (27.9 and 34.8%) tumors, and *vicarious discrimination* and *sexual abuse* were most common among women with HER2 + (39.5 and 34.1%, respectively) and TN (38.7 and 33.4%) young-onset BC. Average adult waist circumference was highest among women diagnosed with TN young-onset BC.

**Table 1:** Weighted distributions of adverse childhood experience (ACE) index and body size characteristics by case/control status and tumor subtype of participants in the Young Women’s Health History Study from Metropolitan Detroit and Los Angeles County^a^

	Controls (N = 1350)	Cases (N = 1754)				
		Overall ^b^	Luminal A	Luminal B	HER2 +	TN
		(N = 1754)	(n = 676)	(n = 549)	(n = 102)	(n = 301)
ACE index score; mean (SE)	2.9 (0.1)	2.7 (0.1)	2.7 (0.1)	2.5 (0.1)	3.0 (0.2)	2.7 (0.1)
	N (W%^c^)	N (W%^c^)	N (W%^c^)	N (W%^c^)	N (W%^c^)	N (W%^c^)
ACE index categories						
0	206 (17.5)	289 (17.3)	113 (18.0)	108 (19.9)	12 (11.3)	40 (13.7)
1	242 (15.7)	326 (19.3)	119 (18.6)	107 (19.7)	15 (15.7)	63 (22.3)
2	244 (18.7)	311 (17.2)	123 (17.5)	98 (17.0)	20 (18.3)	52 (16.8)
> 3	658 (48.1)	828 (46.3)	321 (46.0)	236 (43.4)	55 (54.7)	146 (47.2)
Individual ACEs^d^						
Caregiver illness	168 (16.0)	206 (12.7)	79 (13.6)	60 (11.5)	13 (12.0)	34 (11.8)
Caregiver substance abuse	283 (23.5)	388 (22.5)	173 (25.9)	98 (17.7)	26 (27.9)	58 (19.0)
Police contact ^e^	154 (9.2)	107 (5.6)	39 (5.0)	36 (6.3)	6 (6.0)	19 (5.6)
Caregiver separation	483 (35.0)	606 (33.0)	227 (31.5)	179 (32.0)	36 (35.7)	119 (38.6)
Domestic violence	347 (25.0)	430 (24.0)	197 (27.9)	103 (19.1)	24 (22.7)	70 (22.2)
Death of loved one	311 (19.8)	386 (21.4)	133 (19.1)	117 (20.7)	27 (27.0)	71 (23.2)
Personal discrimination^f^	521 (36.7)	676 (38.4)	263 (38.5)	193 (36.1)	46 (46.0)	121 (39.3)
Vicarious discrimination^f^	549 (33.9)	625 (32.9)	222 (28.8)	182 (32.3)	42 (39.5)	121 (38.7)
Physical abuse	208 (18.9)	267 (15.7)	126 (18.4)	76 (15.5)	17 (15.9)	30 (9.5)
Verbal abuse	478 (37.9)	571 (33.1)	246 (36.2)	162 (30.5)	35 (34.8)	82 (27.3)
Sexual abuse	403 (31.0)	544 (30.4)	202 (28.2)	170 (31.6)	34 (34.1)	101 (33.4)
	**Mean (SE)**^**c**^	**Mean (SE)**^**c**^	**Mean (SE)**^**c**^	**Mean (SE)**^**c**^	**Mean (SE)**^**c**^	**Mean (SE)**^**c**^
Adult BMI (kg/m^2^)^g^	28.5 (0.3)	27.6 (0.2)	27.5 (0.3)	27.2 (0.4)	28.7 (0.9)	28.3 (0.5)
Adult waist circumference (cm)^h^	92.1 (0.5)	91.1 (0.4)	90.5 (0.6)	89.9 (0.7)	92.1 (1.7)	94.5 (1.0)

^a^Excluding participants missing ACE index (n = 34 controls; n = 58 cases)

^b^N = 126 missing tumor subtype

^c^Weighted to the populations of Non-Hispanic Black and Non-Hispanic White young women in Metropolitan Detroit and Los Angeles (LA) County (based on 2010 Census) and account for non-response and case–control matching

^d^Individual ACEs not mutually exclusive; percentages reflect ever exposure to each individual ACE

^e^Police contact includes caregiver incarceration and participant report of negative police interaction

^f^Personal and vicarious experiences of discrimination may be experiences perceived to be motivated by racism, sexism, both racism and sexism, or neither racism nor sexism

^g^BMI 12 months before reference date; for cases this is the date of histologically confirmed breast cancer diagnosis and, for controls, four months prior to completion of YWHHS screening interview. BMI missing for n = 34 cases and n = 38 controls

^h^Waist circumference measured at interview; missing for n = 87 cases and n = 56 controls

*SE* standard error, *TN* triple negative

Table 2 shows the odds of young-onset BC associated with ACEs in fully adjusted models. ACE index was not associated with young-onset BC overall. Odds of HER2 + (e.g., OR = 1.71 [95%CI = 0.88–3.33]) and TN (OR = 1.34 [95%CI = 0.86–2.10]) were elevated among women who reported any ACEs (≥ 1 vs 0), but do not reach significance. In both models, report of 1 ACE (vs 0) was significantly associated with higher odds of TN (e.g., OR = 1.85 [95%CI = 1.08–3.16]), but not other molecular subtypes. There was no evidence that associations between ACE index and young-onset BC varied by subtype (e.g., ≥ 1 vs 0 p_heterogeneity_ = 0.32) nor was there evidence for a linear trend (e.g., all p_trend_ ≥ 0.23). In sensitivity analyses excluding experiences of discrimination, the associations between ACEs and HER2 + and TN were attenuated, primarily for TN (Table S3).

**Table 2:** Fully adjusted and weighted odds of young-onset breast cancer subtypes (versus controls, N = 1350) associated with adverse childhood experiences (ACEs) in the Young Women’s Health History Study

	Young-onset BC overall		Luminal A		Luminal B		HER2 +		TN		*p*_het_^*a*^
	N = 1754		(n = 676)		(n = 549)		(n = 102)		(n = 301)		
	N	Adjusted OR (95% CI)^b^	N	Adjusted OR (95% CI)^b^	N	Adjusted OR (95% CI)^b^	N	Adjusted OR (95% CI)^b^	N	Adjusted OR (95% CI)^b^	
ACE index (range 0–12) ^c^		1.00 (0.95–1.05)		1.01 (0.95–1.07)		0.99 (0.93–1.05)		1.03 (0.94–1.14)		0.96 (0.89- 1.03)	
*p*_*trend*_^d^		*0.93*		*0.83*		*0.77*		*0.47*		*0.23*	
Any vs none											
0	286	REF	112	REF	108	REF	12	REF	38	REF	*0.32*
> 1	1,428	1.17 (0.89–1.53)	569	1.15 (0.83–1.60)	447	1.06 (0.76–1.47)	86	1.71 (0.88–3.33)	251	1.34 (0.86–2.10)	
Below median/median/above median vs none											
0	286	REF	112	REF	108	REF	12	REF	38	REF	*0.63*
1	318	1.34 (0.98–1.83)	118	1.28 (0.86–1.90)	103	1.20 (0.81–1.78)	14	1.52 (0.61–3.78)	61	1.85 (1.08–3.16)*	
2	307	1.01 (0.72–1.41)	123	1.04 (0.70–1.53)	97	0.90 (0.58–1.40)	19	1.58 (0.73–3.38)	50	1.08 (0.62–1.89)	
> 3	803	1.17 (0.87–1.58)	315	1.15 (0.79–1.66)	231	1.07 (0.73–1.55)	51	1.91 (0.93–3.93)	139	1.24 (0.77–1.98)	
ACEs individually (not mutually exclusive; reference = 0 ACEs)^**e**^											
Caregiver illness	22	1.00 (0.64–1.56)	83	0.90 (0.53–1.53)	65	0.98 (0.60–1.60)	14	1.03 (0.36–2.92)	36	1.23 (0.61–2.48)	*0.84*
Caregiver substance abuse	382	1.24 (0.91–1.70)	186	1.31 (0.89–1.94)	105	1.00 (0.67–1.48)	28	2.03 (0.92–4.49)	54	1.16 (0.66–2.05)	*0.27*
Police contact ^f^	102	1.17 (0.65–2.09)	37	1.05 (0.54–2.05)	40	1.37 (0.68–2.75)		-	19	1.33 (0.54–3.25)	*0.71*
Caregiver separation	593	1.07 (0.80–1.44)	236	0.99 (0.69–1.43)	109	0.94 (0.64–1.38)	35	1.70 (0.81–3.55)	121	1.44 (0.91–2.28)	*0.16*
Domestic violence	422	1.22 (0.88–1.69)	204	1.39 (0.92–2.09)	112	0.94 (0.61–1.44)	24	1.62 (0.65–4.01)	67	1.20 (0.69–2.08)	*0.28*
Death of loved one	372	1.37 (0.97–1.94)	139	1.21 (0.77–1.89)	125	1.33 (0.84–2.10)	28	2.50 (1.06–5.88)*	66	1.72 (0.96–3.10)	*0.28*
Personal discrimination^g^	656	1.25 (0.90–1.74)	272	1.28 (0.87–1.88)	201	1.15 (0.75–1.77)	43	1.92 (0.89–4.17)	118	1.20 (0.69–2.08)	*0.56*
Vicarious discrimination^g^	606	1.22 (0.87–1.71)	228	1.08 (0.71–1.72)	188	1.24 (0.81–1.92)	40	1.67 (0.72–3.91)	118	1.36 (0.76–2.42)	*0.73*
Physical abuse	258	1.05 (0.75–1.49)	128	1.10 (0.71–1.73)	79	1.00 (0.62–1.62)	16	1.17 (0.46–2.95)	29	0.74 (0.41–1.31)	*0.68*
Verbal abuse	557	1.11 (0.81–1.51)	256	1.19 (0.81–1.76)	168	0.92 (0.63–1.36)	34	1.44 (0.65–3.21)	80	1.04 (0.63–1.73)	*0.47*
Sexual abuse	529	1.13 (0.80–1.58)	203	1.02 (0.66–1.56)	175	1.18 (0.79–1.76)	37	1.76 (0.82–3.79)	97	1.37 (0.80–2.37)	*0.43*

^a^*p*_*heterogeneity*_ calculated from multinomial regression model adjusted for all covariates

^b^Adjusted for age (years), site (Metropolitan Detroit/LA County), and first-degree family history of breast cancer (yes/no/don’t know), race (non-Hispanic Black/non-Hispanic White), and childhood household socioeconomic position index (continuous)

^c^ACEs: caregiver separation/divorce, death of a loved one, physical abuse, verbal abuse, sexual abuse (sexual harassment, forced sex aged < 18 years), personal experiences of discrimination, vicarious experiences of discrimination, fighting in household, caregiver imprisoned, caregiver substance abuse, negative police interaction, caregiver serious physical/mental illness aged < 13 years

^d^*p*_*trend*_ calculated using continuous ACE index (range 0–12)

^e^Individual ACEs not mutually exclusive (i.e., participants may report multiple ACEs); reference group is participants who reported no ACEs

^f^Police contact includes caregiver incarceration and participant report of negative police interaction

^g^Personal and vicarious experiences of discrimination may be experiences perceived to be motivated by racism, sexism, both racism and sexism, or neither racism nor sexism

*denotes statistical significance at the 5% level; ** indicates cell suppressed due to small sample size

*OR* odds ratio, *CI* confidence interval, *BC* breast cancer, *TN* triple negative

In Table 2, we also report the odds of young-onset BC associated with each individual ACE (vs 0 ACEs), where participants who reported more than one ACE are represented in multiple categories of individual ACEs. Individual ACEs were not associated with young-onset BC overall, and associations did not vary significantly across young-onset BC subtype (all p_heterogeneity_ ≥ 0.16). *Death of a loved one* was significantly associated with HER2 + tumors (OR = 2.21 [95%CI = 1.08–4.52]) and the association with TN young-onset BC approached significance (OR = 1.72 [95%CI = 0.96–3.10]). There was the suggestion of a positive association between some other ACEs and HER2 + young-onset BC (e.g., *caregiver substance abuse*, OR = 2.03 [95%CI = 0.92–4.49]; *personal discrimination*, OR = 1.92 [95%CI = 0.89–4.17]), but estimates were imprecise given limited sample size. Inferences from minimally adjusted models were similar, both for analyses of ACE index and individual ACEs, though the positive association between *vicarious discrimination* and TN tumors reached statistical significance (OR = 1.62 [95%CI = 1.05–2.50]; Table S4). As in models of luminal A and luminal B tumors, no associations with ACEs reached significance when tumor subtype was defined as HR +/HER2- or HR +/HER2 + (data not shown). There was some evidence that having a family history of breast cancer modified the effect of certain ACEs on luminal A tumors (Table S5). The ACEs *caregiver illness*, *domestic violence*, *death of a loved one*, and *sexual abuse* were associated with higher odds of luminal A tumors among women with a family history of BC, but not among women with no family history of BC (p_interaction_ all < 0.02).

Table 3 shows results from the path analyses evaluating the total, indirect (i.e., via adult adiposity: BMI and waist circumference), and direct (i.e., via pathways other than adiposity) effects of ACEs on young-onset BC subtype, also incorporating the potential effects of race and childhood household SEP on ACEs and young-onset BC risk (Fig. 1). The direct effect of ACEs (≥ 1 vs 0) on young-onset BC was significantly positive for all three subtypes, with the strongest association observed for TN tumors (β = 0.132, p < 0.01). Adult BMI and waist circumference only mediated the effect of ACEs for luminal A (−0.009, p = 0.04) and luminal B (−0.014, p < 0.01), where adult adiposity slightly mitigated the effects of ACEs on young-onset BC. The total effect of ACEs on young-onset BC was significantly positive for luminal A (β = 0.079, p = 0.03) and TN (β = 0.131, p < 0.01).

**Table 3:** Standardized estimates of the direct and indirect effect of adverse childhood experiences (ACEs) on young onset breast cancer (BC) subtype risk from weighted path analyses ^a^

	Luminal A	Luminal B	TN
	β (SE)^b^	β (SE)^b^	β (SE)^b^
Direct effect of ACEs (≥ 1 vs 0) on young-onset BC subtype risk ^c^	0.088 (0.038)*	0.081 (0.040)*	0.132 (0.040)*
Total indirect effect of ACEs through BMI and waist circumference on young-onset BC subtype risk ^d^	− 0.009 (0.005)*	− 0.014 (0.005)*	− 0.001 (0.006)
Total effect of ACEs on young-onset BC subtype risk ^e^	0.079 (0.036)*	0.067 (0.038)	0.131 (0.039)*

^a^Separate path analyses conducted for each of the three subtype outcomes (i.e., luminal A, luminal B, and triple negative) vs controls. Analyses not conducted for HER2 + young-onset BC due to limited sample size

^b^Covariates include study site (LA County/Metropolitan Detroit), age at reference date (years, continuous), first degree family history of breast cancer (yes/no/don’t know), race (non-Hispanic Black/non-Hispanic White), and childhood household SEP index (continuous). Mediators included adult body mass index (BMI; m/kg^2^, continuous) and measured adult waist circumference (cm continuous); see Fig. 1

^c^Direct effect of ACEs on young-onset BC risk, accounting for potential mediators and confounders

^d^Indirect effect of ACEs through both BMI and waist circumference

^e^Total effect of ACEs and young-onset BC subtype risk, including effect mediated by adult BMI and waist circumference

*denotes statistical significance at the 5% level

*BC* breast cancer, *TN* triple negative

Table 4 additionally displays the estimated effect of ACEs on adult adiposity and of both ACEs and adult adiposity on young-onset BC subtype from the path analyses. In models of luminal A and TN, ACEs were significantly associated with larger BMI and waist circumference, and BMI was inversely associated with each young-onset BC subtype, though it did not reach significance in the model of luminal B. ACEs were significantly associated with larger BMI but not waist circumference in models of luminal B. Waist circumference was positively associated with all young-onset BC subtypes, though the effect was largest and only reached significance for TN (β = 0.231, p < 0.05). In all three models, the effect of ACEs was larger for BMI than waist circumference.

**Table 4:** Standardized effect estimates of adverse childhood experiences (ACEs) on adult adiposity and of both ACEs and adult adiposity on young onset breast cancer (BC) subtype risk from weighted path analyses ^a, b^

a) Luminal A			
	Adult BMI (per kg/m^2^)	Adult waist circumference (per cm)	Luminal A (vs controls)
*Predictors*	β (SE)	β (SE)	β (SE)
ACEs (≥ 1 vs 0)	0.144 (0.032)*	0.070 (0.016)*	0.088 (0.038)*
Adult BMI		0.788 (0.014)*	− 0.078 (0.028)*
Adult waist circumference			0.011 (0.032)

b) Luminal B			
	Adult BMI (per kg/m^2^)	Adult waist circumference (per cm)	Luminal B (vs controls)
*Predictors*	β (SE)	β (SE)	β (SE)
ACEs (≥ 1 vs 0)	0.192 (0.030)*	0.027 (0.016)	0.081 (0.040)*
Adult BMI		0.785 (0.015)*	− 0.078 (0.044)
Adult waist circumference			0.005 (0.040)

c) Triple negative			
	Adult BMI (per kg/m^2^)	Adult waist circumference (per cm)	TN (vs controls)
*Predictors*	β (SE)	β (SE)	β (SE)
ACEs (≥ 1 vs 0)	0.176 (0.035)*	0.053 (0.017)*	0.132 (0.040)*
Adult BMI		0.774 (0.017)*	− 0.256 (0.039)*
Adult waist circumference			0.231 (0.040)*

^a^Separate path analyses conducted for each of the three subtype outcomes (i.e., luminal A, luminal B, and triple negative) vs controls. Analyses not conducted for HER2 + young-onset BC due to limited sample size

^b^Covariates include study site (LA County/Metropolitan Detroit), age at reference date (years, continuous), first degree family history of breast cancer (yes/no/don’t know), race (non-Hispanic Black/non-Hispanic White), and childhood household SEP index (continuous). Mediators included adult body mass index (BMI; m/kg^2^, continuous) and measured adult waist circumference (cm continuous); see Fig. 1

*denotes statistical significance at the 5% level

*BC* breast cancer, *TN* triple negative

## Discussion

In this first population-based study of ACEs and BC molecular subtypes among young women, experiencing the death of a loved one before age 13 years was associated with more than twice the odds of HER2 + young-onset BC. Reporting one ACE was also associated with nearly twice the odds of TN young-onset BC, but we saw no evidence of a linear trend in associations between number of ACEs and young-onset BC, overall or by subtype. In exploratory path analyses that evaluated adult adiposity as a potential mediator, we additionally observed an association between reporting at least one ACE and both luminal A and TN young-onset BC, where the effect was largest for TN. For luminal A young-onset BC, but not TN tumors, this association was mediated by adult adiposity.

In this study, most individual ACEs were not associated with young-onset BC, and we did not observe evidence of a dose response relationship between number of ACEs and young-onset BC, findings which are generally consistent with prior research. ACE parameterization, BC subtype definitions, and the ages of participants vary between the three prior studies that evaluate these associations [7, 11, 36], however, which complicates comparisons. Consistent with a prior analysis of premenopausal women (N = 337 cases) in the Sister Study cohort, we observed no association between individual ACEs or number of ACEs (i.e., ACE index) and young-onset BC overall [11]. In analyses by ER-status in the full Sister Study cohort (77% postmenopausal at first follow-up), no association was observed between latent class analysis-defined ACE profiles and either ER-positive (N = 1,714) or ER-negative (N = 267) tumors [11]. Inferences in these analyses may have been limited, however, by the small number of cases who experienced any particular ACE or ACE profile (e.g., n = 21 ER-negative Sister Study cases with the profile “sexual trauma and family drug/alcohol/mental health,” n = 3 with “high early life trauma”). In a subtype-specific analysis from the Black Women’s Health Study cohort (77% premenopausal at baseline) [7], experiencing four or more instances of childhood sexual assault (i.e., before age 11 years; vs no childhood sexual or physical abuse) was associated with increased risk of ER-positive BC at any age (HR = 1.35 [95%CI = 1.01–1.79]; n = 55 women with ER-positive BC and childhood sexual abuse). In our study, however, experience of *sexual abuse* before age 18 years was not associated with luminal A tumors (n = 203 exposed cases), and a majority of ER-positive tumors are of the luminal A subtype [60]. In both the Black Women’s Health Study cohort and the present study, childhood physical abuse was not significantly associated with any BC outcome (Black Women’s Health Study, n = 239 ER-positive and n = 120 ER-negative BC cases with physical abuse; YWHHS, n = 16 HER2 + and n = 29 TN young-onset BC cases with physical abuse). In these studies, as in ours, it is possible that the small number of women diagnosed with rarer BC subtypes who had experienced a particular adversity limited our ability to detect effects.

We do observe, however, that *death of a loved one* was significantly associated with 2.5 times higher odds of HER2 + tumors and non-significantly associated with 1.7 times higher odds of TN tumors. It is possible that some of these loved ones were family members who died from breast cancer. However, the positive associations with HER2 + and TN tumors do not appear to be driven by familial BC, as the prevalence of this ACE did not meaningfully differ by family history status, either among cases overall or among cases with HER2 + or TN tumors (data not shown). These findings are also consistent with the one previous study to evaluate the association between parental death before age 21 years and BC diagnosed before age 52 years by tumor subtype [36]. This study, conducted in the hospital-based California Childhood Health and Development Studies cohort, observed that death of both parents (vs no parental death) was significantly if imprecisely associated with increased risk of ER-negative tumors, PR-negative tumors, and HER2-positive tumors, and death of any parent was also significantly associated with HER2-positive tumors (hazard ratio = 3.90 [95%CI = 1.53–9.93]). Small sample size of this rarer HER2 + tumor subtype may limit our ability to detect heterogeneity in associations between *death of a loved one* and young-onset BC subtypes.

We also observe that the effect of ACEs on luminal A tumors was modified by first degree family history of BC, where *caregiver illness*, *domestic violence*, *death of a loved one*, and *sexual abuse* were associated with higher odds among women with a family history but not among those without. Having a family history of BC was not associated with these ACEs, so we do not expect, for example, that the prevalence of *caregiver illness* is driven by caregiver illness from BC. Though the potentially modifying effect of family history has not yet been evaluated in the context of ACEs, there is some evidence that modifiable factors (e.g., parity, alcohol consumption) are associated with higher risk of young-onset BC for women with a first-degree family history of BC compared to those without [61]. It is possible that women with a family history of BC are also more susceptible to the effects of ACEs on young-onset BC risk. Evaluation of these associations in a larger sample of women with rarer BC subtypes would further elucidate the potential role of family history in associations between ACEs and young-onset BC.

In our sample, *personal discrimination* and *vicarious discrimination* were among the most reported ACEs and, consistent with national data, non-Hispanic Black women were more likely than non-Hispanic White women to experience discrimination in childhood [45, 62–64]. Discrimination in adulthood has been associated with increased young-onset BC risk [65], though to our knowledge the association between discrimination in childhood and young-onset BC has not previously been evaluated. In sensitivity analyses omitting both *personal* and *vicarious discrimination* ACEs from the ACE index, the positive association between one ACE and TN young-onset BC was attenuated and became non-significant (Table S4), which suggests that this association may be partly driven by experiences of discrimination. Neither individual discrimination ACE was significantly associated with TN in fully adjusted models, however. Very few non-Hispanic Black women reported no ACEs (7.3% of cases and 7.4% of controls), which curtails our ability to evaluate potential differences in associations between *personal* and *vicarious discrimination* and young-onset BC subtypes by race.

To explore one pathway by which ACEs may affect young-onset BC risk, we additionally evaluated the potential mediating effect of adult adiposity. Consistent with the positive albeit non-significant modest association between ≥ 1 ACEs and young-onset BC subtypes in logistic regression analyses, results from path analyses showed modest positive and significant direct and total effects of ACEs on luminal A and TN young-onset BC. We speculate that the differences in effect scales and test statistics between these two analytic approaches contribute to the differences in statistical significance of the estimates [59, 66]. As expected, we also observed a small negative indirect effect of ACEs via adult adiposity on luminal A young-onset BC where adiposity mitigated the effect of ACEs, given the significant inverse association between increased BMI and luminal A young-onset BC [29, 67]. We did not, however, find evidence to support our hypothesis that adult adiposity positively mediates part of the associations between ACEs and TN young-onset BC, or ACEs and luminal B tumors. For TN tumors, the opposing effects of BMI and waist circumference are of similar magnitude, and thus the indirect effect of ACEs via adult adiposity on TN young-onset BC is negligible. This inverse association that we observe between BMI and TN has been reported in some [67, 68], but not all [30, 69, 70], previous studies of young-onset BC. Additionally, for luminal B tumors, we observe that BMI is non-significantly associated with a small decrease in risk, waist circumference is not meaningfully associated with increased risk, and the indirect effect is small and inverse. The cause for the apparently differing effects of general and central adiposity, measured by BMI and waist circumference, is not yet well understood, though central adiposity is thought to be distinctly pathogenic and may exert a stronger effect on circulating hormones and certain inflammatory markers than general adiposity [71]. Our observation of significant positive direct effects of reporting any ACEs on all three young-onset BC subtypes evaluated—independent of adult adiposity—suggests that other biological pathways may be involved and warrants further investigation.

When interpreting our findings, several limitations should be considered. First, we relied on recalled childhood exposures and adult BMI, which may be subject to recall bias. Previous studies, however, suggest that agreement between prospective assessment and retrospective report of ACEs is higher when data on recalled ACEs is collected through in-person interviews–as was done in the YWHHS–than when collected via self-administered questionnaires [72]. Report of ACEs was also supported by life history calendars, which enhance recall of early life events [44]. Additional measures to increase participant’s comfort in disclosing sensitive information included allowing participants to choose the interview location, matching participants to interviewers based on gender and race/ethnicity, making showcards available for participants to indicate their response nonverbally, and extensive quality control training of interviewers [41]. We cannot rule out the possibility, however, that participants may have underreported ACEs [74–76]. We also note that recalled BMI at interview was highly correlated with measured BMI, and we applied a correction factor to reduce potential bias in recall of BMI 12 months before reference date. While waist circumference was measured at the interview and may have been impacted by young-onset BC or its treatment, evidence suggests that modern treatment regimens are only minimally associated with weight gain [72]. Additionally, because the correlation between measured BMI at interview and recalled BMI 12 months before reference date was high and similar among cases and controls (both r = 0.91, data not shown), we expect that waist circumference prior to diagnosis is similarly correlated with waist circumference after diagnosis. An additional limitation is our use of standard ACE measures, which may inadequately capture the complexity or severity of childhood adversity. The ACE index may obscure distinct risk patterns associated with specific ACEs or their combinations. On the other hand, evaluating ACEs individually may underestimate total adversity [73], because ACEs often co-occur [9]. Our sample size for the rarer young-onset BC subtypes limited our ability to conduct more extensive analyses, including evaluation of multiple mediators, and may also have limited our ability to detect moderate to small associations. We also note that we cannot rule out the possibility of residual confounding by factors such as exposure to environmental pollutants[74] or maternal substance use during pregnancy [75–77], which was likely higher for women with a low childhood household SEP, and which may be implicated in breast cancer risk [78–80].

Our study also has multiple strengths. Our sample is population-based, and findings are generalizable to socioeconomically diverse populations of young non-Hispanic Black and non-Hispanic White women. Additionally, tumor molecular subtypes were classified based on histopathological data from two well-established NCI SEER registries with data on ER, PR, and HER2 status and tumor grade [41]. Including HER2 status improves subtype classification [81], and ER/PR/HER2 information from SEER registries has been shown to be highly valid [82]. We also examine childhood experiences of discrimination, which evidence suggests are important ACEs that affect health [63, 83, 84] yet are infrequently included in studies of ACEs [83, 85]. Because discrimination experiences are especially prevalent among non-Hispanic Black populations—in our sample and nationally [62, 64]—studies that omit discrimination likely underestimate the true burden of adversity borne by Black children. Our study also incorporates in-depth information on childhood SEP, an important health determinant [86–90]. To our knowledge, this study is the first to evaluate potential mediators of associations between ACEs and young-onset BC subtype risk.

Results from this study suggest that experience of bereavement in childhood is associated with an increased risk of HER2 + young-onset BC and that ACEs may also be associated with increased risk of other young-onset BC subtypes, particularly the aggressive TN type. This positive association with TN, however, appears to be mediated through pathways other than adult adiposity. Future studies with larger sample size are needed to explore associations between ACE exposure and young-onset BC subtypes, as well as whether other factors may mediate these associations.

## Supplementary Information

Below is the link to the electronic supplementary material.

Supplementary file1 (DOCX 64 KB)

## Data Availability

Data are available from the corresponding author upon reasonable request and contingent upon approval by appropriate IRBs.

## Abbreviations

YWHHS: Young Women’s Health History Study
ER: Estrogen receptor
PR: Progesterone receptor
HER2: Human epidermal growth factor 2
BC: Breast cancer
OR: Odds ratios
CI: Confidence intervals
ACEs: Adverse childhood experiences
BMI: Body mass index
SEER: Surveillance Epidemiology and End Results Program

## References

[1] Haque AT et al (2023) Cancer mortality rates by racial and ethnic groups in the United States, 2018-2020. J Natl Cancer Inst 115(7):822–830. 10.1093/jnci/djad069 37074947 10.1093/jnci/djad069 PMC10323905

[2] Giaquinto AN et al (2022) Breast cancer statistics, 2022. CA Cancer J Clin 72(6):524–541. 10.3322/caac.21754 36190501 10.3322/caac.21754

[3] Koh B et al (2023) Patterns in cancer incidence among people younger than 50 years in the US, 2010 to 2019. JAMA Netw Open 6(8):e2328171. 10.1001/jamanetworkopen.2023.28171 37585204 10.1001/jamanetworkopen.2023.28171 PMC10433086

[4] Shoemaker M, White M, Wu M, Weir H, Romieu I (2018) Differences in breast cancer incidence among young women aged 20–49 years by stage and tumor characteristics, age, race, and ethnicity, 2004–2013. Breast Cancer Res 169:595–606 10.1007/s10549-018-4699-9 PMC5955792 29445940

[5] Chen HL, Zhou MQ, Tian W, Meng KX, He HF (2016) Effect of age on breast cancer patient prognoses: a population-based study using the SEER 18 database. PLoS ONE 11(10):e0165409. 10.1371/journal.pone.0165409 27798652 10.1371/journal.pone.0165409 PMC5087840

[6] Eccles SA et al (2013) Critical research gaps and translational priorities for the successful prevention and treatment of breast cancer. Breast Cancer Res 15(5):R92. 10.1186/bcr3493 24286369 10.1186/bcr3493 PMC3907091

[7] Barber LE, Zirpoli GR, Cozier YC, Rosenberg L, Petrick JL, Bertrand KA, Palmer JR (2021) Neighborhood disadvantage and individual-level life stressors in relation to breast cancer incidence in US black women. Breast Cancer Res 23(1):108. 10.1186/s13058-021-01483-y 34809694 10.1186/s13058-021-01483-y PMC8609879

[8] Linnenbringer E, Geronimus A, Davis K, Bound J, Ellis L, Gomez S (2020) Associations between breast cancer subtype and neighborhood socioeconomic and racial composition among black and white women. Breast Cancer Res Treat 180(2):437–447 32002766 10.1007/s10549-020-05545-1 PMC7066090

[9] Giano Z, Wheeler DL, Hubach RD (2020) The frequencies and disparities of adverse childhood experiences in the U.S. BMC Public Health 20(1):1327. 10.1186/s12889-020-09411-z 32907569 10.1186/s12889-020-09411-z PMC7488299

[10] Halfon N, Larson K, Son J, Lu M, Bethell C (2017) Income inequality and the differential effect of adverse childhood experiences in US children. Acad Pediatr 17(7S):S70–S78. 10.1016/j.acap.2016.11.007 28865663 10.1016/j.acap.2016.11.007

[11] Woo JMP et al (2022) Latent class models of early-life trauma and incident breast cancer. Epidemiology 33(5):729–738. 10.1097/EDE.0000000000001507 35580243 10.1097/EDE.0000000000001507 PMC9378657

[12] Jacobs JR, Bovasso GB (2000) Early and chronic stress and their relation to breast cancer. Psychol Med 30(3):669–678. 10.1017/s0033291799002020 10883721 10.1017/s0033291799002020

[13] Pino O, Cadena RT, Poli D (2022) A comprehensive review on multifaceted mechanisms involved in the development of breast cancer following adverse childhood experiences (ACEs). Int J Environ Res Public Health. 10.3390/ijerph191912615 36231913 10.3390/ijerph191912615 PMC9565960

[14] Biro FM et al (2013) Onset of breast development in a longitudinal cohort. Pediatrics 132(6):1019–1027. 10.1542/peds.2012-3773 24190685 10.1542/peds.2012-3773 PMC3838525

[15] Eckert-Lind C, Busch AS, Petersen JH, Biro FM, Butler G, Brauner EV, Juul A (2020) Worldwide secular trends in age at pubertal onset assessed by breast development among girls: a systematic review and meta-analysis. JAMA Pediatr 174(4):e195881. 10.1001/jamapediatrics.2019.5881 32040143 10.1001/jamapediatrics.2019.5881 PMC7042934

[16] Rice MS et al (2017) Breast cancer risk prediction: an update to the Rosner-Colditz breast cancer incidence model. Breast Cancer Res Treat 166(1):227–240. 10.1007/s10549-017-4391-5 28702896 10.1007/s10549-017-4391-5 PMC5647223

[17] Rosner B, Colditz GA, Willett WC (1994) Reproductive risk factors in a prospective study of breast cancer: the Nurses’ Health Study. Am J Epidemiol 139(8):819–835. 10.1093/oxfordjournals.aje.a117079 8178795 10.1093/oxfordjournals.aje.a117079

[18] Colditz GA, Bohlke K, Berkey CS (2014) Breast cancer risk accumulation starts early: prevention must also. Breast Cancer Res Treat 145(3):567–579. 10.1007/s10549-014-2993-8 24820413 10.1007/s10549-014-2993-8 PMC4079839

[19] Antonova L, Aronson K, Mueller CR (2011) Stress and breast cancer: from epidemiology to molecular biology. Breast Cancer Res 13(2):208. 10.1186/bcr2836 21575279 10.1186/bcr2836 PMC3219182

[20] Bowen DJ, Fernandez Poole S, White M, Lyn R, Flores DA, Haile HG, Williams DR (2021) The role of stress in breast cancer incidence: risk factors, interventions, and directions for the future. Int J Environ Res Public Health. 10.3390/ijerph18041871 33671879 10.3390/ijerph18041871 PMC7918955

[21] Karnes B, Hanissian A, White BM, Yaun JA, Shaban-Nejad A, Schwartz DL (2025) Exploring the link between adverse childhood experiences and cancer development-insights and intervention recommendations from a scoping review. npj Ment Health Res 4(1):23. 10.1038/s44184-025-00138-6 40456965 10.1038/s44184-025-00138-6 PMC12130255

[22] Wiss DA, Brewerton TD (2020) Adverse childhood experiences and adult obesity: a systematic review of plausible mechanisms and meta-analysis of cross-sectional studies. Physiol Behav 223:112964. 10.1016/j.physbeh.2020.112964 32479804 10.1016/j.physbeh.2020.112964

[23] Thapa K, Shen Y, Cordero JF, Vall EA, Rajbhandari-Thapa J (2025) Associations between adverse childhood experiences and obesity among young US adults. Ann Epidemiol 111:51–57. 10.1016/j.annepidem.2025.09.009 40967352 10.1016/j.annepidem.2025.09.009

[24] Hughes K et al (2017) The effect of multiple adverse childhood experiences on health: a systematic review and meta-analysis. Lancet Public Health 2(8):e356–e366. 10.1016/S2468-2667(17)30118-4 29253477 10.1016/S2468-2667(17)30118-4

[25] Houghton SC, Eliassen H, Tamimi RM, Willett WC, Rosner BA, Hankinson SE (2021) Central adiposity and subsequent risk of breast cancer by menopause status. J Natl Cancer Inst 113(7):900–908. 10.1093/jnci/djaa197 33367714 10.1093/jnci/djaa197 PMC8491796

[26] Chen GC, Chen SJ, Zhang R, Hidayat K, Qin JB, Zhang YS, Qin LQ (2016) Central obesity and risks of pre- and postmenopausal breast cancer: a dose-response meta-analysis of prospective studies. Obes Rev 17(11):1167–1177. 10.1111/obr.12443 27432212 10.1111/obr.12443

[27] Wang F et al (2017) Distinct effects of body mass index and waist/hip ratio on risk of breast cancer by joint estrogen and progestogen receptor status: results from a case-control study in northern and eastern china and implications for chemoprevention. Oncologist 22(12):1431–1443. 10.1634/theoncologist.2017-0148 28912152 10.1634/theoncologist.2017-0148 PMC5728030

[28] Fagherazzi G, Chabbert-Buffet N, Fabre A, Guillas G, Boutron-Ruault MC, Mesrine S, Clavel-Chapelon F (2012) Hip circumference is associated with the risk of premenopausal ER-/PR- breast cancer. Int J Obes (Lond) 36(3):431–439. 10.1038/ijo.2011.66 21427693 10.1038/ijo.2011.66

[29] Post LM et al (2024) Adiposity throughout adulthood and risk of young-onset breast cancer tumor subtypes in the young women’s health history study. Cancer Epidemiol Biomarkers Prev 33(12):1659–1670. 10.1158/1055-9965.EPI-24-1067 10.1158/1055-9965.EPI-24-1067 PMC11609824 39356300

[30] Torres-de la Roche LA, Steljes I, Janni W, Friedl TWP, De Wilde RL (2020) The association between obesity and premenopausal breast cancer according to intrinsic subtypes-a systematic review. Geburtshilfe Frauenheilkd 80(6):601–610. 10.1055/a-1170-5004 32565550 10.1055/a-1170-5004 PMC7299685

[31] van den Brandt PA et al (2021) Body size and weight change over adulthood and risk of breast cancer by menopausal and hormone receptor status: a pooled analysis of 20 prospective cohort studies. Eur J Epidemiol 36(1):37–55. 10.1007/s10654-020-00688-3 33128203 10.1007/s10654-020-00688-3 PMC7847460

[32] M. Eskelinen and P. Ollonen (2010) Life stress and losses and deficit in adulthood as breast cancer risk factor: a prospective case-control study in Kuopio, Finland, *In Vivo,* vol. 24, no. 6, pp. 899–904. [Online]. https://www.ncbi.nlm.nih.gov/pubmed/21164052. 21164052

[33] Surtees PG, Wainwright NW, Luben RN, Khaw KT, Bingham SA (2010) No evidence that social stress is associated with breast cancer incidence. Breast Cancer Res Treat 120(1):169–174. 10.1007/s10549-009-0454-6 19572196 10.1007/s10549-009-0454-6

[34] Wise LA, Palmer JR, Boggs DA, Adams-Campbell LL, Rosenberg L (2011) Abuse victimization and risk of breast cancer in the black women’s health study: abuse and breast cancer risk in black women. Cancer Causes Control 22(4):659–669. 10.1007/s10552-011-9738-3 21327459 10.1007/s10552-011-9738-3 PMC3153377

[35] Boddy AM, Rupp S, Yu Z, Hanson H, Aktipis A, Smith K (2022) Early life adversity, reproductive history and breast cancer risk. Evol Med Public Health 10(1):429–438. 10.1093/emph/eoac034 36101671 10.1093/emph/eoac034 PMC9464099

[36] Keeler C, Krigbaum NY, Cohn B, Cirillo P (2025) Parental loss at age birth to 21 years and daughters’ breast cancer and tumor characteristics. JNCI Cancer Spectr. 10.1093/jncics/pkaf004 39820352 10.1093/jncics/pkaf004 PMC11892429

[37] Chan DSM et al (2019) World cancer research fund international: continuous update project-systematic literature review and meta-analysis of observational cohort studies on physical activity, sedentary behavior, adiposity, and weight change and breast cancer risk. Cancer Causes Control 30(11):1183–1200. 10.1007/s10552-019-01223-w 31471762 10.1007/s10552-019-01223-w

[38] Millikan RC et al (2008) Epidemiology of basal-like breast cancer. Breast Cancer Res Treat 109(1):123–139. 10.1007/s10549-007-9632-6 17578664 10.1007/s10549-007-9632-6 PMC2443103

[39] McCarthy AM et al (2021) Relationship of established risk factors with breast cancer subtypes. Cancer Med 10(18):6456–6467. 10.1002/cam4.4158 34464510 10.1002/cam4.4158 PMC8446564

[40] Li C, Fan Z, Lin X, Cao M, Song F, Song F (2021) Parity and risk of developing breast cancer according to tumor subtype: a systematic review and meta-analysis. Cancer Epidemiol 75:102050. 10.1016/j.canep.2021.102050 34706325 10.1016/j.canep.2021.102050

[41] Velie EM et al (2021) Theory, methods, and operational results of the young women’s health history study: a study of young-onset breast cancer incidence in black and white women. Cancer Causes Control 32(10):1129–1148. 10.1007/s10552-021-01461-x 34292440 10.1007/s10552-021-01461-x PMC8416838

[42] AAPOR (2016) Standard definitions: Final dispositions of case codes and outcome rates for surveys

[43] Overview of the SEER Program (2024) National Cancer Institute. https://seer.cancer.gov/about/overview.html

[44] Wingo PA, Ory HW, Layde PM, Lee NC (1988) The evaluation of the data collection process for a multicenter, population-based, case-control design. Am J Epidemiol 128(1):206–217. 10.1093/oxfordjournals.aje.a114942 3381827 10.1093/oxfordjournals.aje.a114942

[45] Post LM et al (2025) Associations between childhood socioeconomic characteristics, race, and risk of adverse childhood experiences in a population-based sample of US-born non-Hispanic Black and White women. BMC Public Health 25(1):1636. 10.1186/s12889-025-22589-4 10.1186/s12889-025-22589-4 PMC12046649 40316955

[46] Centers for Disease Control and Prevention (CDC) (2009) Behavioral risk factor surveillance system survey questionnaire. ed. Atlanta, Georgia: U.S. Department of Health and Human Services, Centers for Disease Control and Prevention

[47] Dominguez TP, Dunkel-Schetter C, Glynn LM, Hobel C, Sandman CA (2008) Racial differences in birth outcomes: the role of general, pregnancy, and racism stress. Health Psychol 27(2):194–203. 10.1037/0278-6133.27.2.194 18377138 10.1037/0278-6133.27.2.194 PMC2868586

[48] Martel P, Mbofana F, Cousens S (2021) The polychoric dual-component wealth index as an alternative to the DHS index: addressing the urban bias. J Glob Health 11:04003. 10.7189/jogh.11.04003 33643634 10.7189/jogh.11.04003 PMC7897450

[49] Vyas S, Kumaranayake L (2006) Constructing socio-economic status indices: how to use principal components analysis. Health Policy Plan 21(6):459–468. 10.1093/heapol/czl029 17030551 10.1093/heapol/czl029

[50] Lee RD, Chen J (2017) Adverse childhood experiences, mental health, and excessive alcohol use: examination of race/ethnicity and sex differences. Child Abuse Negl 69:40–48. 10.1016/j.chiabu.2017.04.004 28448813 10.1016/j.chiabu.2017.04.004 PMC5896758

[51] Houtepen LC, Heron J, Suderman MJ, Fraser A, Chittleborough CR, Howe LD (2020) Associations of adverse childhood experiences with educational attainment and adolescent health and the role of family and socioeconomic factors: a prospective cohort study in the UK. PLoS Med 17(3):e1003031. 10.1371/journal.pmed.1003031 32119668 10.1371/journal.pmed.1003031 PMC7051040

[52] Hirko KA et al (2024) Lifetime alcohol consumption patterns and young-onset breast cancer by subtype among non-hispanic black and white women in the young women’s health history study. Cancer Causes Control 35(2):377–391. 10.1007/s10552-023-01801-z 37787924 10.1007/s10552-023-01801-z

[53] Ihenacho U et al (2022) lifetime personal cigarette smoking and risk of young-onset breast cancer by subtype among non-hispanic black and white women in the young women’s health history study. Breast Cancer Res Treat 195(3):353–366. 10.1007/s10549-022-06675-4 35925453 10.1007/s10549-022-06675-4 PMC10424682

[54] Williams K, Finch BK (2019) Adverse childhood experiences, early and nonmarital fertility, and women’s health at midlife. J Health Soc Behav 60(3):309–325. 10.1177/0022146519868842 31526017 10.1177/0022146519868842

[55] Maguire-Jack K, Sattler K (2023) Neighborhood poverty, family economic well-being, and child maltreatment. J Interpers Violence 38(5–6):4814–4831. 10.1177/08862605221119522 36062823 10.1177/08862605221119522 PMC10276351

[56] Maguire-Jack K, Lanier P, Lombardi B (2020) Investigating racial differences in clusters of adverse childhood experiences. Am J Orthopsychiatry 90(1):106–114. 10.1037/ort0000405 30816722 10.1037/ort0000405

[57] Clarke CA, Keegan TH, Yang J, Press DJ, Kurian AW, Patel AH, Lacey JV Jr. (2012) Age-specific incidence of breast cancer subtypes: understanding the black-white crossover. J Natl Cancer Inst 104(14):1094–1101. 10.1093/jnci/djs264 22773826 10.1093/jnci/djs264 PMC3640371

[58] Akinyemiju TF, Demb J, Izano MA, Rehkopf DH, Fang ML, Hiatt RA, Braithwaite D (2018) The association of early life socioeconomic position on breast cancer incidence and mortality: a systematic review. Int J Public Health 63(7):787–797. 10.1007/s00038-017-1060-8 29197969 10.1007/s00038-017-1060-8 PMC5984656

[59] Muthen LK, Muthen BO (2017) Mplus user’s guide (1998-2017), Eigth Edition ed. Muthen & Muthen, Los Angeles, CA

[60] American Cancer Society (2024) Breast cancer facts & figures 2024–2025. Atlanta

[61] O’Brien KM et al (2025) Pathogenic variants, family history, and cumulative risk of breast cancer in US women. JAMA Oncol 11(12):1458–1469. 10.1001/jamaoncol.2025.3875 41066089 10.1001/jamaoncol.2025.3875 PMC12512027

[62] Bleich SN et al (2019) Discrimination in the united states: experiences of black Americans. Health Serv Res 54(Suppl 2):1399–1408. 10.1111/1475-6773.13220 31663124 10.1111/1475-6773.13220 PMC6864380

[63] Williams DR, Lawrence JA, Davis BA (2019) Racism and health: evidence and needed research. Annu Rev Public Health 40(1):105–125. 10.1146/annurev-publhealth-040218-043750 30601726 10.1146/annurev-publhealth-040218-043750 PMC6532402

[64] Lee RT, Perez AD, Boykin CM, Mendoza-Denton R (2019) On the prevalence of racial discrimination in the united states. PLoS ONE 14(1):e0210698. 10.1371/journal.pone.0210698 30629706 10.1371/journal.pone.0210698 PMC6328188

[65] Taylor TR et al (2007) Racial discrimination and breast cancer incidence in US Black women: the black women’s health study. Am J Epidemiol 166(1):46–54. 10.1093/aje/kwm056 17400570 10.1093/aje/kwm056

[66] Stata Base Reference Manual: Release 16, ed. College Station, Texas: StataCorp, LLC (2019)

[67] G. Premenopausal Breast Cancer Collaborative et al (2018) Association of body mass index and age with subsequent breast cancer risk in premenopausal women. JAMA Oncol 4(11):e181771. 10.1001/jamaoncol.2018.1771 29931120 10.1001/jamaoncol.2018.1771 PMC6248078

[68] Ma H et al (2018) Body mass index at age 18 years and recent body mass index in relation to risk of breast cancer overall and ER/PR/HER2-defined subtypes in white women and African-American women: a pooled analysis. Breast Cancer Res 20(1):5. 10.1186/s13058-017-0931-5 29357906 10.1186/s13058-017-0931-5 PMC5778748

[69] Fortner RT, Katzke V, Kuhn T, Kaaks R (2016) Obesity and breast cancer. Recent Results Cancer Res 208:43–65. 10.1007/978-3-319-42542-9_3 27909901 10.1007/978-3-319-42542-9_3

[70] Chen L, Cook LS, Tang MT, Porter PL, Hill DA, Wiggins CL, Li CI (2016) Body mass index and risk of luminal, HER2-overexpressing, and triple negative breast cancer. Breast Cancer Res Treat 157(3):545–554. 10.1007/s10549-016-3825-9 27220749 10.1007/s10549-016-3825-9 PMC5575777

[71] Elks CM, Francis J (2010) Central adiposity, systemic inflammation, and the metabolic syndrome. Curr Hypertens Rep 12(2):99–104. 10.1007/s11906-010-0096-4 20424938 10.1007/s11906-010-0096-4

[72] van den Berg MM et al (2017) Weight change during chemotherapy in breast cancer patients: a meta-analysis. BMC Cancer 17(1):259. 10.1186/s12885-017-3242-4 28403873 10.1186/s12885-017-3242-4 PMC5389147

[73] Lacey RE, Howe LD, Kelly-Irving M, Bartley M, Kelly Y (2022) The clustering of adverse childhood experiences in the avon longitudinal study of parents and children: are gender and poverty important? J Interpers Violence 37(5–6):2218–2241. 10.1177/0886260520935096 32639853 10.1177/0886260520935096 PMC8918866

[74] Mohai P, Lantz PM, Morenoff J, House JS, Mero RP (2009) Racial and socioeconomic disparities in residential proximity to polluting industrial facilities: evidence from the americans’ changing lives study. Am J Public Health 99(Suppl 3):S649–S656. 10.2105/AJPH.2007.131383 19890171 10.2105/AJPH.2007.131383 PMC2774179

[75] Brenner AB, Diez Roux AV, Barrientos-Gutierrez T, Borrell LN (2015) Associations of alcohol availability and neighborhood socioeconomic characteristics with drinking: cross-sectional results from the multi-ethnic study of atherosclerosis (MESA). Subst Use Misuse 50(12):1606–1617. 10.3109/10826084.2015.1027927 26579610 10.3109/10826084.2015.1027927 PMC4802501

[76] Hill TD, Angel RJ (2005) Neighborhood disorder, psychological distress, and heavy drinking. Soc Sci Med 61(5):965–975. 10.1016/j.socscimed.2004.12.027 15955398 10.1016/j.socscimed.2004.12.027

[77] Moss JL, Pinto CN, Shen C (2025) Prevalence of cancer risk behaviors by county-level persistent poverty. Cancer Epidemiol 94:102735. 10.1016/j.canep.2024.102735 39709835 10.1016/j.canep.2024.102735

[78] Madrigal JM et al (2024) Carcinogenic industrial air pollution and postmenopausal breast cancer risk in the national institutes of health aarp diet and health study. Environ Int 191:108985. 10.1016/j.envint.2024.108985 39226766 10.1016/j.envint.2024.108985 PMC11425761

[79] VoPham T et al (2020) Dioxin exposure and breast cancer risk in a prospective cohort study. Environ Res 186:109516. 10.1016/j.envres.2020.109516 32305677 10.1016/j.envres.2020.109516 PMC7363533

[80] Cogliano VJ et al (2011) Preventable exposures associated with human cancers. J Natl Cancer Inst 103(24):1827–1839. 10.1093/jnci/djr483 22158127 10.1093/jnci/djr483 PMC3243677

[81] Anderson WF, Rosenberg PS, Katki HA (2014) Tracking and evaluating molecular tumor markers with cancer registry data: HER2 and breast cancer. J Natl Cancer Inst. 10.1093/jnci/dju093 24777110 10.1093/jnci/dju093 PMC4677264

[82] Ma H et al (2009) Breast cancer receptor status: do results from a centralized pathology laboratory agree with SEER registry reports? Cancer Epidemiol Biomarkers Prev 18:2214–2220 19661080 10.1158/1055-9965.EPI-09-0301 PMC3782852

[83] Bernard DL, Calhoun CD, Banks DE, Halliday CA, Hughes-Halbert C, Danielson CK (2021) Making the ‘“C-ACE”’ for a culturally-informed adverse childhood experiences framework to understand the pervasive mental health impact of racism on black youth. J Child Adolesc Trauma 14(2):233–247. 10.1007/s40653-020-00319-9 33986909 10.1007/s40653-020-00319-9 PMC8099967

[84] Miller HN, Perrin N, Thorpe RJ Jr., Evans MK, Zonderman AB, Allen J (2022) The association between perceived discrimination and BMI trajectory: a prospective study of African American and white adults. Fam Community Health 45(3):206–213. 10.1097/FCH.0000000000000326 35385415 10.1097/FCH.0000000000000326 PMC9156529

[85] Currie CL, Copeland JL, Metz GA (2019) Childhood racial discrimination and adult allostatic load: the role of Indigenous cultural continuity in allostatic resiliency. Soc Sci Med 241:112564. 10.1016/j.socscimed.2019.112564 31605950 10.1016/j.socscimed.2019.112564

[86] Parolin Z, Matsudaira J, Waldfogel J, Wimer C (2022) Exposure to childhood poverty and racial differences in economic opportunity in young adulthood. Demography 59(6):2295–2319. 10.1215/00703370-10350740 36409157 10.1215/00703370-10350740

[87] Do DP, Zheng C (2017) A marginal structural modeling strategy investigating short and long-term exposure to neighborhood poverty on BMI among U.S. black and white adults. Health Place 46:201–209. 10.1016/j.healthplace.2017.05.010 28551568 10.1016/j.healthplace.2017.05.010

[88] Kwarteng JL, Schulz AJ, Mentz GB, Israel BA, Shanks TR, Perkins DW (2016) Neighbourhood poverty, perceived discrimination and central adiposity in the USA: independent associations in a repeated measures analysis. J Biosoc Sci 48(6):709–722. 10.1017/S0021932016000225 27238086 10.1017/S0021932016000225 PMC5800399

[89] Bleich SN, Thorpe RJ Jr., Sharif-Harris H, Fesahazion R, Laveist TA (2010) Social context explains race disparities in obesity among women. J Epidemiol Community Health 64(5):465–469. 10.1136/jech.2009.096297 20445215 10.1136/jech.2009.096297 PMC3099623

[90] Phelan BLJ (1995) Social conditions as fundamental causes of disease. J Health Soc Behav. 10.2307/2626958 7560851

