# Supplementary material for: Adverse childhood experiences, adult adiposity, and risk of young-onset breast cancer subtypes in a population-based case-control study
Source: Cancer Causes Control. 2026 May 26;37(6):97. doi: 10.1007/s10552-026-02171-y (PMC13212395; doi:10.1007/s10552-026-02171-y)
Supplement: Supplementary file 1 — Supplementary file1 (DOCX 64 KB) [file 10552_2026_2171_MOESM1_ESM.docx]

**Table S1. Description of adverse childhood experiences (ACEs) in the Young Women’s Health History Study.**

| **ACE name** | **Definition of each ACE and age assessed** |
| --- | --- |
| *Caregiver illness* | Parent/primary caregiver had a serious physical or mental illness that affected family life or their ability to work. Assessed for age <13 years; response options were yes/no. |
| *Caregiver substance abuse* | Parent/primary caregiver neglected their responsibilities, family, or work for two or more days in a row because of alcohol drinking or drug use, or their drinking or drug use created problems between them and family members. Assessed for age <13 years; response options were yes/no. |
| *Caregiver incarceration* | Parent/primary caregiver served time in a prison, jail, or other correctional facility. Assessed for age <13 years; response options were yes/no. |
| *Caregiver separation* | Parents/primary caregivers had gotten separated or divorced. Assessed for age <13 years; response options were yes/no. |
| *Domestic violence* | Parents/caregivers/adults in home ever slapped, hit, kicked, punched, or beaten up on another. Assessed for age <13 years; response options were 0, 1, 2-5, 6-10, 11-20, 21-50, >50 times. |
| *Death of loved one* | Experienced the death of someone close, such as a parent, primary caregiver, brother, or sister. Assessed for age <13 years; response options were yes/no. |
| *Police interaction* | Picked up, arrested, taken away, or harassed by the police. Assessed for age <13 years; response options were yes/no. |
| *Personal discrimination* ^a^ | Experienced discrimination/unfair treatment in public, in school by staff, in school by peers, or in situations with police or legal authorities. Assessed for age <13 years; see footnote for response option details. |
| *Vicarious discrimination* ^a^ | Witnessed or heard about discrimination/unfair treatment experienced by a close loved one in public, job-related situations, or situations with police or legal authorities. Assessed for age <13 years; see footnote for response option details. |
| *Physical abuse* | Parent/caregiver/adult in home ever hit, beat, kicked, or physically hurt the participant (excluding spanking). Assessed for age <13 years; response options were 0, 1, 2-5, 6-10, 11-20, 21-50, >50 times. |
| *Verbal abuse* | Parent/caregiver/adult in home called them names, swore at them, or put them down. Assessed for age <13 years; response options were 0, 1, 2-5, 6-10, 11-20, 21-50, >50 times. |
| *Sexual abuse* | An adult or someone >5 years older touched them sexually or tried to make the participant touch them sexually OR experienced forced sex, including any type of penetration or intercourse where verbal threats, pressure, physical force, or a weapon is used. It also includes situations where they could not give consent because of being a minor, under the influence of drugs or alcohol, or asleep. Assessed for age <18 years and age <13 years; all sexual abuse (i.e., any sexual abuse reported age <18 years) included in the present analyses. |

^a^  Participants reported whether they or their loved ones encountered discrimination or unfair treatment in these settings often, sometimes, rarely, or never. Weighted latent class analysis was used to identify two levels of discrimination exposure (“higher” and “lower”) and two levels of vicarious discrimination exposure (“higher” and “lower”); these two binary variables were used in analyses. These classes have previously been described in detail (see ^45^).

| **Table S2. Distribution of sociodemographic characteristics among participants with and without adverse childhood experiences (ACE) index values, by case/control status** | | | | | | |
| --- | --- | --- | --- | --- | --- | --- |
|  | **Cases** | | | **Controls** | | |
|  | **Not missing ACEs** | **Missing at least one ACE** |  | **Not missing any ACEs** | **Missing at least one ACE** |  |
|  | **N=1,754 (96.8%)** | **N=58**  **(3.2%)** |  | **N=1,350**  **(97.0%)** | **N=31**  **(3.0%)** |  |
|  | **N (W%** ^a^**)** | **N (W%** ^a^**)** | ***X^2^ p-value*** | **N (W%** ^a^**)** | **N (W%** ^a^**)** | ***X^2^ p-value*** |
| Age at reference date ^b^ |  |  | *0.56* |  |  | *0.01* |
| 20-29 years | 2.9 | 1.7 |  | 2.9 | 2.4 |  |
| 30-39 years | 19.4 | 15.5 |  | 19.7 | 5.8 |  |
| 40-49 years | 77.7 | 82.8 |  | 77.4 | 91.9 |  |
| Birth cohort |  |  | *0.60* |  |  | *0.11* |
| Born 1961-1965 | 25.1 | 31.1 |  | 26.2 | 49.2 |  |
| Born 1966-1975 | 60.7 | 56.8 |  | 58.2 | 45.2 |  |
| Born 1976-1985 | 13.1 | 12.0 |  | 13.8 | 3.8 |  |
| Born 1986-1995 | 21.2 | 0.0 |  | 1.8 | 1.8 |  |
| Study Site |  |  | *0.38* |  |  | *0.35* |
| Metropolitan Detroit | 53.1 | 59.4 |  | 53.7 | 40.0 |  |
| Los Angeles County | 46.9 | 40.6 |  | 46.3 | 60.0 |  |
| Racial identity |  |  | *0.18* |  |  | *0.39* |
| Non-Hispanic Black | 29.9 | 38.0 |  | 30.4 | 22.7 |  |
| Non-Hispanic White | 70.1 | 62.0 |  | 69.6 | 77.3 |  |
| Childhood household SEP index |  |  | *<0.01* |  |  | *<0.01* |
| Higher | 47.6 | 22.6 |  | 42.6 | 11.4 |  |
| Medium | 21.4 | 17.1 |  | 21.4 | 2.3 |  |
| Lower | 29.0 | 24.8 |  | 33.9 | 63.1 |  |
| Missing | 2.1 | 35.5 |  | 2.1 | 23.3 |  |
|  |  |  |  |  |  |  |
|  | **Weighted Mean (SE)** | **Weighted Mean (SE)** | ***T-test p-value^c^*** | **Weighted Mean (SE)** | **Weighted Mean (SE)** | ***T-test p-value^c^*** |
| Adult BMI (kg/m^2^) | 27.6 (0.2) | 28.0 (0.87) | *0.63* | 28.5 (0.3) | 30.0 (1.8) | *0.31* |
| Adult waist circumference (cm) | 91.1 (0.4) | 91.0 (2.7) | *0.59* | 92.1 (0.5) | 94.6 (3.5) | *0.57* |

^a^ Weighted to the populations of Non-Hispanic Black and Non-Hispanic White young women in Metropolitan Detroit and Los Angeles (LA) County (based on 2010 Census) and account for non-response and case-control matching. Column percentages add to 100.

^b^ The reference date is, for cases this is the date of histologically confirmed breast cancer diagnosis and, for controls, four months prior to completion of YWHHS screening interview.

| **Table S3. Adjusted and weighted odds of young-onset breast cancer subtypes associated with adverse childhood experiences (ACEs) – excluding experiences of discrimination— in the Young Women’s Health History Study** | | | | | | | | | | | | | | | | | | | | | | | | | | | | |
| --- | --- | --- | --- | --- | --- | --- | --- | --- | --- | --- | --- | --- | --- | --- | --- | --- | --- | --- | --- | --- | --- | --- | --- | --- | --- | --- | --- | --- |
|  | **Controls** | **Overall cases** | | | | | **Luminal A** | | | | | | **Luminal B** | | | | | | **HER2+** | | | | | **TN** | | | | *p_heterogeneity_^a^* |
|  | **N=1,350** | **N=1,754** | | | | | **N=676** | | | | | | **N=549** | | | | | | **N=102** | | | | | **N=301** | | | |  |
|  | **N** | **N** | **Minimally adjusted** | | **Fully adjusted** | | **N** | | **Minimally adjusted1** | | **Fully adjusted** | | **N** | | **Minimally adjusted** | | **Fully adjusted** | | **N** | **Minimally adjusted** | | | **Fully adjusted** | **N** | **Minimally adjusted** | | **Fully adjusted** |  |
|  |  |  | **OR**  **(95% CI)^b^** | | **OR**  **(95% CI)^c^** | |  |  | **OR**  **(95% CI)^b^** | | **OR**  **(95% CI)^c^** | |  |  | **OR**  **(95% CI)^b^** | | **OR**  **(95% CI)^c^** | |  | **OR**  **(95% CI)^b^** | | | **OR**  **(95% CI)^c^** |  | **OR**  **(95% CI)^b^** | | **OR**  **(95% CI)^c^** |  |
| **ACE index (range 0-10) ^d^** | | | | | | | | | | | | | | | | | | | | | | | | | | | | |
| *p_trend_*^e^ |  |  | *0.09* | | *0.63* | |  | | *0.42* | | *0.97* | |  | | *0.04* | | *0.52* | |  | *0.96* | | | *0.88* |  | *0.11* | | *0.16* |  |
| **Any vs none** | | | | | | | | | | | | | | | | | | | | | | | | | | | | |
| 0 | 316 | 424 | REF | | REF | | 165 | | REF | | REF | | 146 | | REF | | REF | | 19 | REF | | | REF | 70 | REF | | REF | *0.69* |
| >1 | 996 | 1,294 | 1.01  (0.81-1.25) | | 1.10  (0.88-1.37) | | 504 | | 0.96  (0.73-1.27) | | 1.06  (0.79-1.41) | | 395 | | 0.97  (0.74-1.26) | | 1.10  (0.84-1.44) | | 77 | 1.56  (0.91-2.66) | | | 1.54  (0.87-2.74) | 219 | 1.07  (0.76-1.50) | | 1.03  (0.74-1.45) |  |
|  |  |  | |  | |  | |  | |  | |  | |  | |  | |  | | |  |  | |  | |  | |  |
| **Below median/median/above median vs none** | | | | | | | | | | | | | | | | | | | | | | | | | | | | |
| 0 | 316 | 424 | REF | | REF | | 165 | | REF | | REF | | 146 | | REF | | REF | | 19 | REF | | | REF | 70 | REF | | REF | *0.71* |
| 1 | 328 | 426 | 1.15  (0.90-1.46) | | 1.16  (0.90-1.50) | | 154 | | 1.02  (0.75-1.39) | | 1.09  (0.79-1.50) | | 141 | | 1.20  (0.88-1.63) | | 1.22  (0.87-1.67) | | 21 | 1.47  (0.73-2.95) | | | 1.36  (0.65-2.83) | 80 | 1.36  (0.90-2.07) | | 1.25  (0.83-1.87) |  |
| 2 | 213 | 308 | 1.01  (0.74-1.39) | | 1.05  (0.76-1.46) | | 116 | | 0.96  (0.64-1.43) | | 1.00  (0.66-1.51) | | 102 | | 1.04  (0.72-1.51) | | 1.11  (0.76-1.63) | | 21 | 1.87  (0.92-3.80) | | | 1.85  (0.91-3.76) | 45 | 0.93  (0.56-1.55) | | 0.89  (0.54-1.48) |  |
| >3 | 455 | 560 | 0.92  (0.71-1.19) | | 1.06  (0.81-1.39) | | 234 | | 0.93  (0.67-1.29) | | 1.07  (0.75-1.53) | | 152 | | 0.79  (0.56-1.12) | | 0.98  (0.68-1.41) | | 35 | 1.47  (0.82-2.63) | | | 1.49  (0.75-2.97) | 94 | 0.95  (0.65-1.40) | | 0.95  (0.63-1.42) |  |

^a^ *p_heterogeneity_* calculated from multinomial regression model adjusted for Model 2 covariates.

^b^ Adjusted for age (years), site (Metropolitan Detroit/LA County), and first-degree family history of breast cancer (yes/no/don’t know).

^c^ Adjusted for age (years), site (Metropolitan Detroit/LA County), and first-degree family history of breast cancer (yes/no/don’t know), race (non-Hispanic Black/non-Hispanic White), and childhood household socioeconomic position index (continuous).

^d^ ACEs including caregiver separation or divorce, death of a loved one, physical abuse, verbal abuse, sexual abuse (sexual harassment or forced sex before age 18 years), fighting in household, caregiver imprisoned, caregiver substance abuse, negative police interaction, and caregiver serious physical/mental illness before age 13 years.

^e^ *p_trend_* calculated using continuous ACE index (range 0-10).

| **Table S4. Minimally adjusted and weighted odds of young-onset breast cancer subtypes (versus controls, N=1,350) associated with adverse childhood experiences (ACEs) in the Young Women’s Health History Study** | | | | | |
| --- | --- | --- | --- | --- | --- |
|  | **Young-onset**  **BC** **overall** | **Luminal A** | **Luminal B** | **HER2+** | **TN** |
|  | N=1,754 | (n=676) | (n=549) | (n=102) | (n=301) |
|  | **Minimally adjusted**  **OR (95% CI)^a^** | **Minimally adjusted**  **OR (95% CI)^a^** | **Minimally adjusted**  **OR (95% CI)^a^** | **Minimally adjusted**  **OR (95% CI)^a^** | **Minimally adjusted**  **OR (95% CI)^a^** |
| **ACE index (range 0-12) ^b^** | 0.97 (0.93-1.01) | 0.97 (0.92-1.03) | 0.95 (0.89-1.00) | 1.03 (0.96-1.11) | 0.97 (0.92-1.03) |
| *p_trend_*^c^ | *0.17* | *0.38* | *0.07* | *0.45* | *0.37* |
| **Any vs none** | | | | | |
| 0 | REF | REF | REF | REF | REF |
| >1 | 1.07 (0.83-1.38) | 1.02 (0.74-1.40) | 0.92 (0.68-1.25) | 1.77 (0.95-3.31) | 1.45 (0.96-2.19) |
|  |  |  |  |  |  |
| **Below median/median/above median vs none** | | | | | |
| 0 | REF | REF | REF | REF | REF |
| 1 | 1.31 (0.96-1.77) | 1.22 (0.83-1.79) | 1.17 (0.81-1.71) | 1.59 (0.65-3.87) | 1.93 (1.17-3.18)* |
| 2 | 0.97 (0.70-1.34) | 0.96 (0.66-1.39) | 0.85 (0.55-1.29) | 1.61 (0.77-3.36) | 1.24 (0.72-2.14) |
| >3 | 1.02 (0.78-1.35) | 0.97 (0.69-1.38) | 0.86 (0.61-1.21) | 1.91 (1.00-3.62) | 1.37 (0.90-2.11) |
| **ACEs individually (not mutually exclusive; reference=0 ACEs) ^d^** | | | | | |
| Caregiver illness | 0.86 (0.58-1.28) | 0.82 (0.51-1.33) | 0.75 (0.48-1.18) | 1.33 (0.51-3.47) | 1.18 (0.61-2.27) |
| Caregiver substance abuse | 1.00 (0.76-1.32) | 1.08 (0.75-1.55) | 0.72 (0.50-1.04) | 1.94 (0.98-3.81) | 1.13 (0.70-1.85) |
| Police contact ^e^ | 0.71 (0.47-1.06) | 0.62 (0.36-1.07) | 0.76 (0.47-1.23) | - | 0.89 (0.45-1.74) |
| Caregiver separation | 0.95 (0.72-1.26) | 0.88 (0.62-1.26) | 0.77 (0.55-1.09) | 1.58 (0.81-3.08) | 1.46 (0.97-2.20) |
| Domestic violence | 1.01 (0.75-1.36) | 1.12 (0.76-1.64) | 0.72 (0.49-1.06) | 1.55 (0.72-3.37) | 1.21 (0.77-1.90) |
| Death of loved one | 1.11 (0.81-1.52) | 0.92 (0.61-1.37) | 1.00 (0.67-1.49) | 2.21(1.08-4.52)* | 1.64 (0.99-2.73) |
| Personal discrimination ^f^ | 1.10 0.82-1.46) | 1.04 (0.73-1.48) | 0.94 (0.66-1.33) | 1.92 (0.96-3.84) | 1.49 (0.95-2.34) |
| Vicarious discrimination ^f^ | 1.03 (0.79-1.35) | 0.86 (0.59-1.24) | 0.92 (0.66-1.29) | 1.99 (0.99-4.02) | 1.62 (1.05-2.50)* |
| Physical abuse | 0.84 (0.61-1.16) | 0.93 (0.61-1.42) | 0.72 (0.47-1.12) | 1.18 (0.50-2.78) | 0.71 (0.41-1.25) |
| Verbal abuse | 0.90 (0.68-1.20) | 0.95 (0.65-1.39) | 0.74 (0.52-1.04) | 1.40 (0.70-2.83) | 0.99 (0.63-1.56) |
| Sexual abuse | 1.00 (0.75-1.34) | 0.87 (0.59-1.28) | 0.95 (0.68-1.33) | 1.73 (0.84-3.56) | 1.49 (0.92-2.40) |
| ^a^ Adjusted for age (years), site (Metropolitan Detroit/LA County), and first-degree family history of breast cancer (yes/no/don’t know).  ^b^ ACEs: caregiver separation/divorce, death of a loved one, physical abuse, verbal abuse, sexual abuse (sexual harassment, forced sex aged <18 years), personal experiences of discrimination, vicarious experiences of discrimination, fighting in household, caregiver imprisoned, caregiver substance abuse, negative police interaction, caregiver serious physical/mental illness aged <13 years.  ^c^ *p_trend_* calculated using continuous ACE index (range 0-12)  ^d^Individual ACEs not mutually exclusive (i.e., participants may report multiple ACEs); reference group is participants who reported no ACEs.  ^e^ Police contact includes caregiver incarceration and participant report of negative police interaction.  ^f^ Personal and vicarious experiences of discrimination may be experiences perceived to be motivated by racism, sexism, both racism and sexism, or neither racism nor sexism.  ***** denotes statistical significance at the 5% level.  OR: odds ratio; CI: confidence interval; BC: breast cancer; TN: triple negative | | | | | |

| **Table S5. Fully adjusted and weighted odds of young-onset breast cancer subtypes associated with adverse childhood experiences (ACEs), with effect modification by first degree family history of breast cancer, in the Young Women’s Health History Study** | | | | | | | | | | |  |
| --- | --- | --- | --- | --- | --- | --- | --- | --- | --- | --- | --- |
|  | **Young-onset**  **BC** **overall** | | **Luminal A** | | **Luminal B** | | **HER2+^a^** | | **TN** | | |
|  | N=1,723 | | (n=663) | | (n=546) | | (n=94) | | (n=296) | | |
|  | **N** | **Fully adjusted**  **OR (95% CI) ^b^** | **N** | **Fully adjusted**  **OR (95% CI) ^b^** | **N** | **Fully adjusted**  **OR (95% CI) ^b^** | **N** | **Fully adjusted**  **OR (95% CI) ^b^** | **N** | **Fully adjusted**  **OR (95% CI) ^b^** | |
| **Any^c^ vs none [Ref=0]** |  |  |  |  |  |  |  |  |  |  | |
| No 1° family history | 1,093 | 1.09 (0.81-1.47) | 412 | 1.01 (0.70-1.44) | 344 | 1.01 (0.70-1.48) | 63 | 1.73 (0.69-4.30) | 199 | 1.35 (0.81-2.24) | |
| Has 1° family history | 272 | 1.66 (0.94-2.93) | 120 | 2.03 (1.06-3.91)* | 72 | 1.15 (0.52-2.54) | 15 | 3.03 (0.52-17.70) | 38 | 1.41 (0.58-3.48) | |
| *p_interaction_^d^* |  | *0.18* |  | *0.05* |  | *0.78* |  |  |  | *0.95* | |
| **Below median/median/above median vs none [Ref=850]** | | | | |  |  |  |  |  |  | |
| No 1° family history |  |  |  |  |  |  |  |  |  |  | |
| 1 | 242 | 1.22 (0.87-1.71) | 87 | 1.10 (0.72-1.70) | 81 | 1.12 (0.71-1.76) | 9 | - | 49 | 1.84 (1.02-3.34) | |
| 2 | 239 | 0.98 (0.68-1.42) | 98 | 1.01 (0.66-1.56) | 75 | 0.85 (0.52-1.37) | 12 | 1.45 (0.51-4.11) | 42 | 1.18 (0.62-2.24) | |
| >3 | 612 | 1.08 (0.77-1.51) | 227 | 0.96 (0.64-1.44) | 188 | 1.05 (0.69-1.61) | 42 | 2.06 (0.78-5.45) | 108 | 1.21 (0.71-2.07) | |
| Has 1° family history |  |  |  |  |  |  |  |  |  |  | |
| 1 | 60 | 2.02 (0.88-4.61) | 26 | 2.40 (0.98-5.90) | 19 | 1.56 (0.55-4.47) | ** | - | 8 | - | |
| 2 | 57 | 1.14 (0.54-2.42) | 23 | 1.30 (0.57-2.96) | 16 | 0.80 (0.30-2.21) | ** | - | 7 | - | |
| >3 | 155 | 1.78 (0.99-3.20) | 71 | 2.25 (1.11-4.58) | 37 | 1.17 (0.51-2.70) | 7 | - | 23 | - | |
| *p_interaction_^d^* |  | *0.43* |  | *0.12* |  | *0.90* |  |  |  | *0.86* | |
| **ACEs individually (not mutually exclusive; reference=0 ACEs) ^e^** | | | | |  |  |  |  |  |  | |
| Caregiver illness |  |  |  |  |  |  |  |  |  |  | |
| No 1° family history | 664 | 0.82 (0.49-1.39) | 135 | 0.62 (0.33-1.17) | 49 | 0.93 (0.53-1.63) | 10 | - | 24 | 1.07 (0.47-2.42) | |
| Has 1° family history | 162 | 1.95 (0.93-4.06) | 25 | 2.61 (1.09-6.26)* | 13 | 1.12 (0.38-3.31) | ** | - | 9 | - | |
| *p_interaction_^d^* |  | *0.05* |  | *0.01** |  | *0.76* |  |  |  | *0.45* | |
| Caregiver substance abuse |  |  |  |  |  |  |  |  |  |  | |
| No 1° family history | 922 | 1.17 (0.82-1.66) | 127 | 1.12 (0.72-1.75) | 82 | 1.10 (0.70-1.74) | 22 |  | 45 | 1.27 (0.67-2.38) | |
| Has 1° family history | 200 | 1.57 (0.81-3.07) | 42 | 2.14 (0.98-4.68) | 16 | 0.71 (0.27-1.84) | 6 | - | 7 | - | |
| *p_interaction_^d^* |  | *0.42* |  | *0.14* |  | *0.43* |  |  |  | *0.39* | |
| Caregiver separation |  |  |  |  |  |  |  |  |  |  | |
| No 1° family history | 1288 | 1.02 (0.74-1.42) | 173 | 0.90 (0.61-1.34) | 147 | 0.92 (0.59-1.44) | 30 |  | 97 | 1.47 (0.86-2.51) | |
| Has 1° family history | 234 | 1.23 (0.67-2.25) | 48 | 1.47 (0.68-3.16) | 27 | 0.81 (0.35-1.88) | ** | - | 17 | 1.23 (0.50-3.02) | |
| *p_interaction_^d^* |  | *0.57* |  | *0.24* |  | *0.78* |  |  |  | *0.72* | |
| Domestic violence |  |  |  |  |  |  |  |  |  |  | |
| No 1° family history | 1008 | 1.11 (0.76-1.60) | 137 | 1.14 (0.73-1.79) | 84 | 0.90 (0.55-1.47) | 18 |  | 56 | 1.28 (0.68-2.44) | |
| Has 1° family history | 208 | 1.83 (0.93-3.58) | 51 | 2.78 (1.23-6.27)* | 18 | 0.98 (0.39-2.46) | ** | - | 9 | - | |
| *p_interaction_^d^* |  | *0.16* |  | *<0.01** |  | *0.86* |  |  |  | *0.62* | |
| Death of loved one |  |  |  |  |  |  |  |  |  |  | |
| No 1° family history | 943 | 1.17 (0.82-1.67) | 90 | 0.86 (0.55-1.36) | 100 | 1.28 (0.78-2.10) | 23 |  | 50 | 1.46 (0.80-2.67) | |
| Has 1° family history | 182 | 3.02 (1.27-7.22)* | 34 | 4.09 (1.50-11.17)* | 14 | 1.41 (0.43-4.58) | ** | - | 11 | 2.63 (0.81-8.55) | |
| *p_interaction_^d^* |  | *0.04** |  | *<0.01** |  | *0.88* |  |  |  | *0.35* | |
| Personal discrimination ^f^ |  |  |  |  |  |  |  |  |  |  | |
| No 1° family history | 1344 | 1.20 (0.82-1.76) | 189 | 1.14 (0.73-1.78) | 158 | 1.16 (0.71-1.90) | 35 |  | 92 | 1.27 (0.70-2.32) | |
| Has 1° family history | 261 | 1.53 (0.78-2.98) | 60 | 2.02 (0.94-4.35) | 31 | 1.08 (0.43-2.72) | 6 | - | 18 | 1.14 (0.38-3.39) | |
| *p_interaction_^d^* |  | *0.54* |  | *0.19* |  | *0.89* |  |  |  | *0.85* | |
| Vicarious discrimination ^f^ |  |  |  |  |  |  |  |  |  |  | |
| No 1° family history | 1338 | 1.18 (0.82-1.68) | 166 | 1.03 (0.66-1.62) | 140 | 1.16 (0.74-1.83) | 13 |  | 94 | 1.41 (0.75-2.66) | |
| Has 1° family history | 248 | 1.66 (0.82-3.35) | 47 | 1.68 (0.70-4.02) | 33 | 1.58 (0.61-4.11) | 7 | - | 19 | 1.48 (0.54-4.05) | |
| *p_interaction_^d^* |  | *0.35* |  | *0.27* |  | *0.52* |  |  |  | *0.92* | |
| Physical abuse |  |  |  |  |  |  |  |  |  |  | |
| No 1° family history | 753 | 0.98 (0.65-1.48) | 84 | 0.92 (0.55-1.55) | 60 | 0.98 (0.56-1.73) | 13 |  | 25 | 0.87 (0.45-1.70) | |
| Has 1° family history | 164 | 1.26 (0.59-2.68) | 28 | 1.66 (0.65-4.22) | 15 | 1.04 (0.38-2.90) | ** | - | ** | - | |
| *p_interaction_^d^* |  | *0.56* |  | *0.26* |  | *0.92* |  |  |  | *0.28* | |
| Verbal abuse |  |  |  |  |  |  |  |  |  |  | |
| No 1° family history | 1225 | 1.02 (0.73-1.45) | 176 | 1.01 (0.65-1.57) | 126 | 0.86 (0.56-1.33) | 27 |  | 67 | 1.15 (0.64-2.04) | |
| Has 1° family history | 242 | 1.48 (0.78-2.80) | 58 | 2.06 (0.95-4.45) | 32 | 1.09 (0.47-2.51) | ** | - | 10 | - | |
| *p_interaction_^d^* |  | *0.29* |  | *0.09* |  | *0.62* |  |  |  | *0.57* | |
| Sexual abuse |  |  |  |  |  |  |  |  |  |  | |
| No 1° family history | 1139 | 0.99 (0.68-1.44) | 139 | 0.82 (0.51-1.30) | 31 | 1.06 (0.68-1.64) | 31 |  | 75 | 1.28 (0.69-2.35) | |
| Has 1° family history | 223 | 1.83 (0.96-3.52) | 48 | 2.22 (1.01-4.88)* | 25 | 1.40 (0.56-3.52) | ** | - | 18 | 1.69 (0.63-4.52) | |
| *p_interaction_^d^* |  | *0.07* |  | *0.02** |  | *0.56* |  |  |  | *0.59* | |
| ^a^ Estimates for HER2+ tumors not presented because sample size was inadequate to estimate associations for women with a family history of BC, rendering comparison with estimates among women with no family history impossible.  ^b^ Adjusted for age (years), site (Metropolitan Detroit/LA County), first-degree family history of breast cancer (yes/no), race (non-Hispanic Black/non-Hispanic White), and childhood household socioeconomic position index (continuous) as well as an interaction term between the exposure and first-degree family history of breast cancer (yes/no). ORs (95% CIs) represent the conditional association between each exposure and outcome within strata of first degree family history of breast cancer (yes/no) derived using linear combination of coefficients.  ^c^ ACEs: caregiver separation/divorce, death of a loved one, physical abuse, verbal abuse, sexual abuse (sexual harassment, forced sex aged <18 years), personal experiences of discrimination, vicarious experiences of discrimination, fighting in household, caregiver imprisoned, caregiver substance abuse, negative police interaction, caregiver serious physical/mental illness aged <13 years.  ^d^ *p_interaction_* calculated using a Wald test of the interaction term between first degree family history (yes/no) and each exposure in the fully adjusted model.  ^e^ Individual ACEs not mutually exclusive (i.e., participants may report multiple ACEs); reference group is participants who reported no ACEs.  ^f^  Personal and vicarious experiences of discrimination may be experiences perceived to be motivated by racism, sexism, both racism and sexism, or neither racism nor sexism.  ***** denotes statistical significance at the 5% level; ** indicates cells suppressed due to small sample size.  OR: odds ratio; CI: confidence interval; BC: breast cancer; TN: triple negative | | | | | | | | | | |  |
